# A Predictive Model of Intein Insertion Site for Use in the Engineering of Molecular Switches

**DOI:** 10.1371/journal.pone.0037355

**Published:** 2012-05-23

**Authors:** James Apgar, Mary Ross, Xiao Zuo, Sarah Dohle, Derek Sturtevant, Binzhang Shen, Humberto de la Vega, Philip Lessard, Gabor Lazar, R. Michael Raab

**Affiliations:** Agrivida Inc., Medford, Maryland, United States of America; University of Lethbridge, Canada

## Abstract

Inteins are intervening protein domains with self-splicing ability that can be used as molecular switches to control activity of their host protein. Successfully engineering an intein into a host protein requires identifying an insertion site that permits intein insertion and splicing while allowing for proper folding of the mature protein post-splicing. By analyzing sequence and structure based properties of native intein insertion sites we have identified four features that showed significant correlation with the location of the intein insertion sites, and therefore may be useful in predicting insertion sites in other proteins that provide native-like intein function. Three of these properties, the distance to the active site and dimer interface site, the SVM score of the splice site cassette, and the sequence conservation of the site showed statistically significant correlation and strong predictive power, with area under the curve (AUC) values of 0.79, 0.76, and 0.73 respectively, while the distance to secondary structure/loop junction showed significance but with less predictive power (AUC of 0.54). In a case study of 20 insertion sites in the XynB xylanase, two features of native insertion sites showed correlation with the splice sites and demonstrated predictive value in selecting non-native splice sites. Structural modeling of intein insertions at two sites highlighted the role that the insertion site location could play on the ability of the intein to modulate activity of the host protein. These findings can be used to enrich the selection of insertion sites capable of supporting intein splicing and hosting an intein switch.

## Introduction

Native intein coding sequences are elements found inserted in-frame into their host gene. Post-translationally, the intein domain is capable of splicing out of the host, in either cis- or trans-splicing reactions, connecting the amino(N-)- and carboxy(C-)-terminal residues of the host exteins resulting in an active mature protein [1], [2], [3]. Frequently, the intein containing precursor protein is inactive prior to splicing, and then activated upon splicing [4]. Based on the observed splicing-dependent changes in host activity, using regulated intein splicing to control the activity of the mature protein can be an effective tool for creating molecular switches. Inteins have been transferred into a number of different host proteins and engineered to control their activity. Proteins have been regulated with varying stimuli such as light [5], small molecules [6], [7], [8], [9] and temperature [4], [10].

Inteins are not universally transferable between proteins. There are specific characteristics for intein insertion sites that allow functional splicing [11], [12], [13], [14], [15]. This has been demonstrated by several experiments including one study where different portions of the intein’s native exteins are transferred with the intein [15], and mutagenesis studies that have shown the preference for the local sequence context [16], preference for particular flanking extein residues [15], [17], [18], [19] and the preference for inteins to splice out of some but not all sites in a target protein [11], [12], [13], [14], [15], [20].

The specificity of sites for both intein splicing and host activation is understandable given the number of interconnected events that must occur in this process. First, the intein must be located in a site that allows adequate protein expression, stability, and folding, preventing intracellular degradation. Second, if the intein regulates the host protein’s activity, the intein insertion must reversibly block the activity of its protein host. This could in principle occur in a number of different ways including blocking the access of the substrate to the active site, altering the local structure of the active site, or interfering with dimerization required to form the active complex. Finally, the local extein environment must allow the intein to efficiently splice out of the host protein [21], [22], [23], and leave a stable, properly folded mature protein with native-like activity. The complex mechanism of intein splicing is seamlessly conducted by native intein/extein pairs. Therefore, if it is possible to identify the important properties of sites that are capable of hosting an intein in their native environment, we can utilize this information to identify functional insertion sites in proteins that do not otherwise host inteins, and aid the development of regulated inteins that act as molecular switches. Our ultimate goal is to develop regulated inteins to control activity of specific enzymes that are detrimental to a desired host organism’s growth and development [24], and express them in the desired host.

In this work we examined features of native inteins to determine which features are highly correlated with the location of the intein insertion site. These include sequence and structure based properties of the intein’s flanking extein, such as location of insertion sites in conserved sequence region of the host, sequence similarities of the splice site cassette, local secondary structure, flexibility of the intein insertion site, burial of the insertion site and distance of the insertion site to the active site and the dimer interface. Of these, conservation of position, local sequence, local secondary structure and distance to both the active site and dimer interface were seen to be statistically significant predictors of the insertion site, while the flexibility of the insertion site and the degree of burial were not significant. Additionally, the conservation of the site, local sequence and distance to the active site/dimer interface were shown to have strong predictive power in identifying native splice sites, while the predictive strength of the local secondary structure was somewhat weaker. Finally, to see if this method could be used to insert inteins into a non-native host protein, we looked at a case study of 20 insertion sites in the XynB xylanase.

## Results

### Analysis of Intein Native Insertion Sites

**Figure 1:**
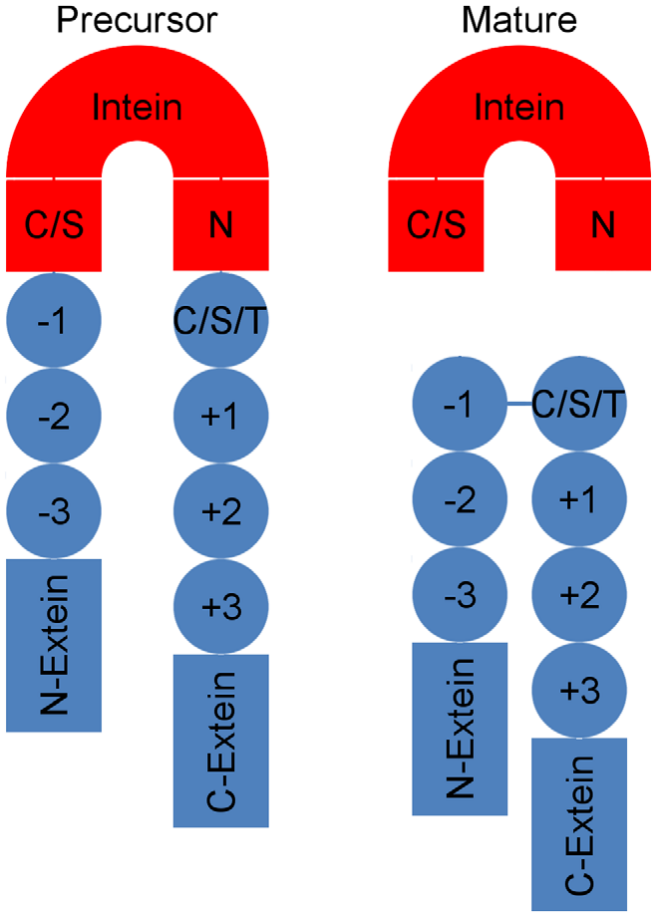
Schematic diagram of precursor and mature intein modified protein. The precursor intein modified protein consists of N- and C-extein domains (blue) broken up by an intein insertion domain (red). Active inteins predominately contain a conserved N-terminal cysteine or serine residue (C/S) and a conserved C-terminal asparagine (N). The extein site where the intein is inserted is typically N-terminal to a cysteine, serine, or threonine residue (C/S/T). The extein residues surrounding the intein insertion site (−3 to +4) are designated as the intein insertion site cassette. Upon splicing the protein forms the mature form and the −1 and C/S/T residues at the +1 are covalently joined.

To analyze the properties of native inteins, flanking extein sequences were extracted (Table S1) from a pruned version of the intein database current as of June 2, 2009 (http://www.neb.com/neb/inteins.html); [25], [26], [27]. The local six amino acids surrounding the intein insertion site (XXX-[cysteine/serine/threonine]-XXX) was defined as the intein insertion site cassettes (Figure 1). Additionally, seven residue “decoy” cassettes were also extracted from these extein sequences in which the center position (corresponding to the +1 position of native insertion sites) was a cysteine, serine or threonine, but the site was not a confirmed intein insertion site. Non-annotated splice sites were designated as non-splice sites since native inteins are not completely transferrable [11], [12], [13], [14], [15]. Thus this set may contain some false negatives.

Using this set of exteins and cassettes, sequence and structural properties were evaluated to determine which had a strong correlation with the location of the intein insertion site. These properties were analyzed by evaluating the characteristics of native insertion sites in relation to the characteristics of all sites that contain a cysteine, serine or threonine (C/S/T sites). The properties evaluated were the conservation of the position of intein insertion site, the sequence similarity of native cassettes, the local secondary structure, the flexibility of the site, the degree of burial and the distance of the insertion site to the active site and dimer interface.

### Location of Insertion Sites to Conserved Sites

**Figure 2:**
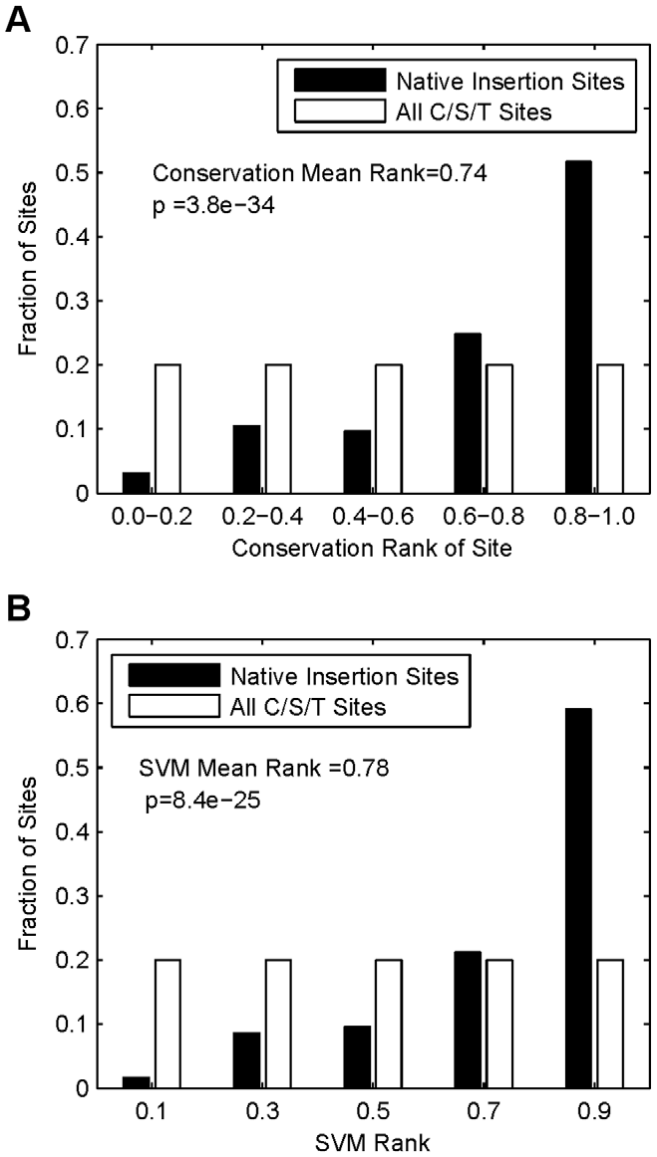
Sequence based native-like features of intein insertion sites. Native intein insertion sites (black bars) were compared to all C/S/T sites (white bars) to determine if there was a significant difference in several features. (**a**) Conservation of intein insertion sites using positional entropy calculated from a PSSM. (**b**) Ranking of native intein insertion sites cassettes using an SVM model.

Conservation among intein insertion regions within the exteins has been previously reported through the analysis of small numbers of inteins [28], [29], [30], as well as for aligned extein families [31]. This is thought to be an important property of the insertion site that would allow for the intein to remain through evolutionary drift [32]. The relative conservation of intein insertion sites versus all other native cysteine/serine/threonine sites was determined by using a sequence entropy method (Methods). Sites with low entropy in general are more conserved than sites with higher entropy. To normalize this dataset, for each extein the native intein insertion site was ranked against all C/S/T sites within the extein. This ranking score was given as a value between 0 and 1, where 0 would be the least conserved C/S/T site (highest entropy) and 1 the most conserved (lowest entropy). A distribution of the rank scores for the native insertion sites was compared to the distribution of all sites using the Wilcoxon rank sum test (Figure 2a). The native sites show a significantly different distribution from all other C/S/T extein sites. The mean rank of the native sites was 0.74 and had a p-value of 3.8×10^−34^. This clearly indicates that the insertion sites occur in more conserved positions that the average C/S/T site.

### Sequence Similarities of Splice Site Cassettes

We used a support vector machine (SVM) to evaluate the similarity of all C/S/T sites to native insertion sites. The support vectors describe the intein insertion site cassettes in vector form. The positive training set was selected from the set of native intein insertion site cassettes. These were filtered for redundancy. Additionally, three random, non-native, “decoy” cassettes were also selected from each extein with a cassette in the positive test set. Each set of three cassettes was selected so as to not include duplicates. The SVM was trained using SVMlight [33], [34] (Methods). To test the SVM we performed a leave-one-out cross validation. Here for a given target cassette the SVM was trained only on positive and negative cassettes from other exteins. We scored all the sites in the target extein, and rank-ordered the native intein insertion site in relation to the remaining sites. This was repeated 25 times for each site and repeated for all exteins. The distribution of average ranks for each native insertion site is shown in Figure 2b. This distribution is significantly different from the distribution for all the non-native sites, with a mean value of 0.78 and a p-value of 8.4×10^−25^.

### Homology Models of Exteins

In order to determine structural information about the intein insertion site environment, homology models of the exteins were generated. Extein sequences were modeled onto structures in the Protein Data Bank [35] with high sequence homology (Methods). This set of structures was filtered to remove sequence redundancy and to include only those where the native intein-insertion site was present.

### Secondary Structure Location

**Figure 3:**
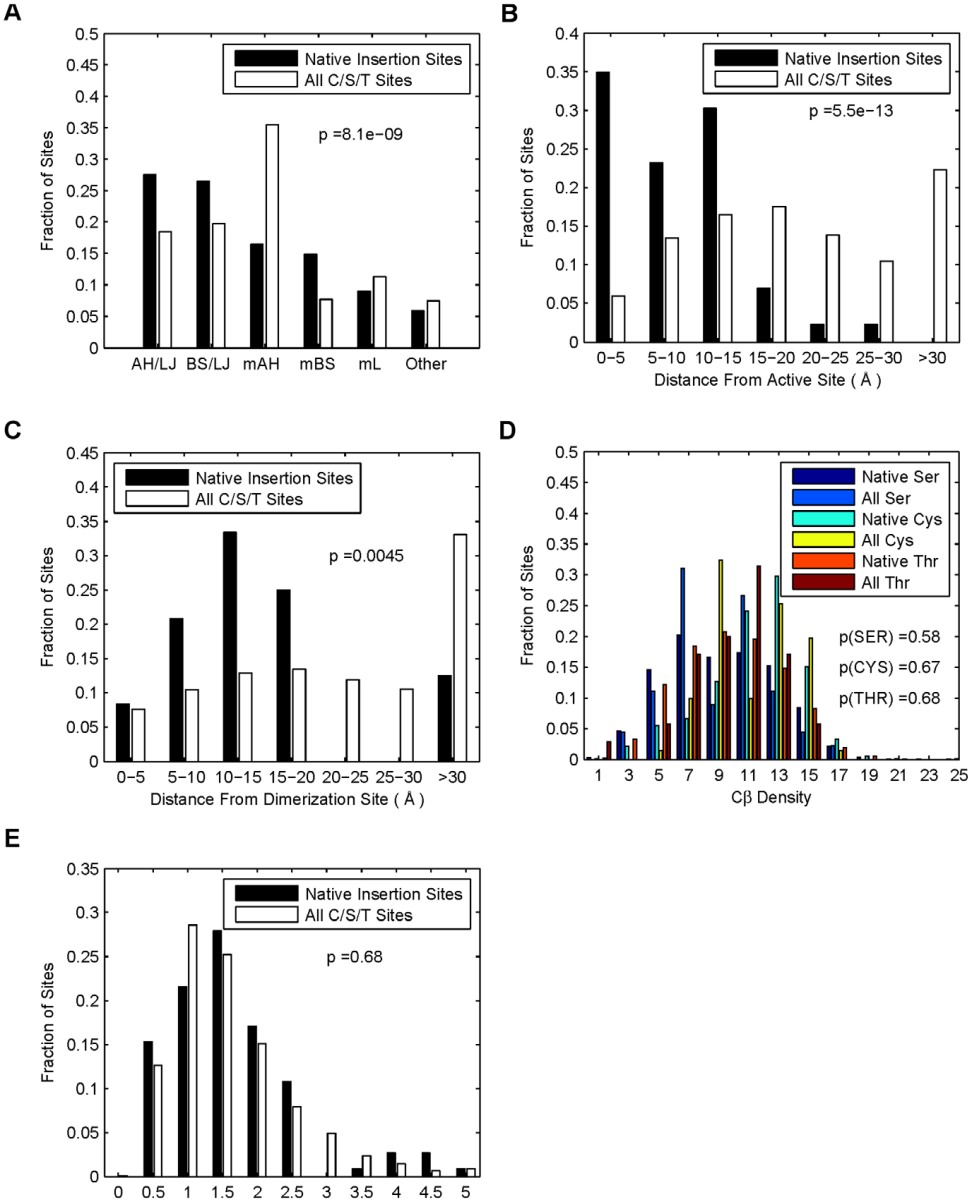
Structure based native-like features of intein insertion sites. (**a**) Local secondary structure of intein insertion sites binned into 6 different groups relative to its location to loop/alpha-helix and loop/beta-sheet junctions. These groups are AH/L-J (within 2 amino acids of an alpha-helix/Loop junction), BS/L-J (within 2 amino acids of an beta-sheet/Loop Junction), mid-AH (middle of an alpha helix and more than two amino acids to a loop junction), mid-BS (middle a beta-sheet and more than two amino acids to a loop junction) mid-L (middle of a loop and more than 2 amino acids to a loop or beta-sheet junction, and other (all remaining sites). (**b–c**) Proximity of intein insertion sites to the closest (b) active site residue or (c) dimer interface residue. (**d**) Degree of burial of intein insertion sites divided by C/S/T residue type. The degree of burial was calculated using the C_β_ density. (**e**) Flexibility of the insertion site residue as determined by RMSD of molecular dynamics simulation. This plot shows the flexibility rank of the native versus non-native insertion sites.

Using the homology models, the local secondary structure of all residues was evaluated using the program Stride [36]. For all C/S/T sites, the proximity of these to α-helix/loop and β-sheet/loop junctions was determined. Each site was binned into one of the following six groups: 1) within two amino acids of an α-helix/loop junction, 2) within 2 amino acids of an β-sheet/loop junction, 3) middle of an α-helix defined as more than two amino acids to a loop junction, 4) middle of a β-sheet defined as more than two amino acids to a loop junction, 5) middle of a loop defined as more than two amino acids to a alpha-helix or beta-sheet junction, and 6) all other classifications. Figure 3a shows a comparison of the distribution of native to non-native C/S/T sites, which are seen to be significantly different by chi-squared analysis (p-value of 8×10^−9^). Further analysis showed that native intein insertion sites are more likely to occur close to a loop/β-sheet or loop/α-helix boundary (54% of sites), than to occur in the middle of a β-sheet or an α-helix (31% of sites). For all C/S/T sites these values are 38% and 43% respectively (Figure 3a). This difference gives a p-value of 10^−5^ using a chi-squared test. Moreover, the native sites are more likely to occur at or near the end of a β-sheet than at or near the end of a α-helix. The ratio of α-helix to β-sheet sites is 41.2% to 43.9% for native-insertion sites, and 53.9% to 27.4% for all C/S/T sites. This is also a significant difference (p-value of 4×10^−5^) using a chi-squared test.

### Proximity of Active Site and Dimer Interface Residues to Native Insertion Sites

The proximity of the intein insertion site to two different types of functionally important residues, active site residues and dimer interface residues of split inteins was measured in our analysis. Of the homology models described above, 43 models were identified with annotated active site residues and 24 with annotated dimer interface residues. These annotations were derived from homologous sequences in the NCBI genpept database. We then measured the distance of the C_α_ position of the native intein insertion site to the closest identified active site or dimer interface residue. Measurements were also carried out for all C/S/T sites. These distances were binned into groups of 5 Å and shown in Figure 3b, c. The mean distance for the native sites to active site residues was 8.3 Å and that for the remaining C/S/T sites was 21.1. The difference of the native and non-native distributions was determined to be significant using the Wilcoxon rank sum test (significance value of 10^−13^). Similarly for the dimer interface site, the native insertion sites are on average closer to the dimer interface sites than the average C/S/T sites. These were shown to have a mean distance of 16.6 to 24.1 Å respectively and have a significance value of 0.0045.

### Degree of Burial

The degree of burial for each intein insertion site was examined and compared with the degree of burial for random C/S/T sites using a method of C_β_-density. This method has been used to describe the burial environment of sites in different applications [37], [38] and can determine whether a position is in the core, boundary or exposed position of a protein. The C_β_-density was calculated as the number of C_β_ atoms within 8 Å of a particular site. For each amino acid type of cysteine, serine and threonine, the native insertion sites were compared to the remainder of the extein sites. For each amino acid type the distribution of the C_β_-densities for native intein insertion sites is not significantly different from the random sites. These distributions are shown in Figure 3d.

### Flexibility of Insertion Site Residues

The relative flexibility of the insertion site may be an important issue when determining sites that are amenable to intein insertion. Here we investigated relative flexibility at the insertion site by performing molecular dynamic equilibration simulations on our set of homology models as described in the methods. Briefly, each model was solvated into a TIP3 water sphere that had a radius that was 5 Å larger than the protein in each dimension. These were minimized for 1000 steps and run with equilibrium molecular dynamics for 5 ns in steps of 2 fs. Snapshots of this trajectory were taken every 50 ps. For the last 50 (2.5 ns) snapshots the time-averaged root mean square deviation of the C_α_-atom position of all C/S/T sites were calculated.

The distribution of native sites is compared to the distribution of all other C/S/T sites in this data set and presented in Figure 3e. These two distributions had similar mean values, 1.296 and 1.285 Å respectively. Using the Wilcoxon rank sum test these were shown to not be significantly different distributions with a P-value of 0.7.

### Analysis of Predictive Value

Of the features tested, four showed significant correlation with the location of the intein insertion site. To determine the predictive quality of these features, first the true positive rate (TPR) (sensitivity) and the false positive rate (FPR) (1-specificity) were calculated for each feature as shown in the following equation. The cutoff point above which a target would be predicted as a positive and below which it would be predicted as a negative was optimized. For each cutoff, the true positives (TP), true negatives (TN), false positives (FP) and false negatives (FN) were counted and used to calculate the TPR, and the FPR.
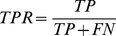
(1)

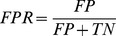
(2)


**Figure 4:**
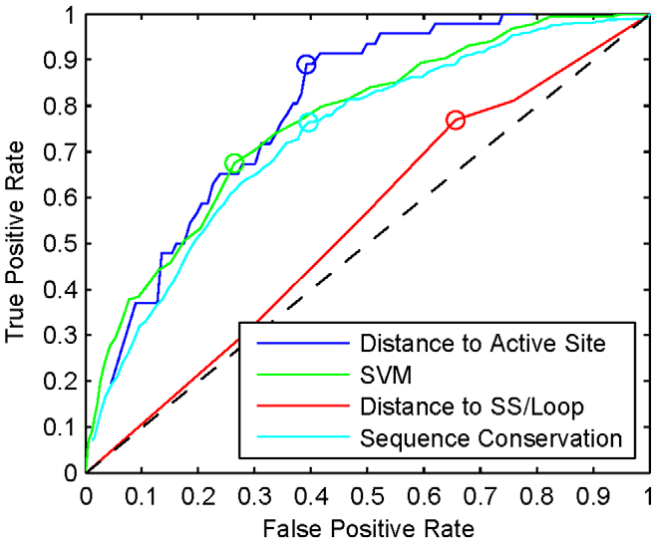
ROC curve of predictive features for intein insertion sites. The true positive rate and the false positive rate was calculated for the 4 features: (blue) distance to the active site or dimer interface, (green) SVM score, (red) distance to SS/Loop junction and (cyan) sequence conservation. These rates were determined over a range of cutoff values which were: for the distance to the active site or dimer interface site the cutoff value was varied from 0 to 30 Å, for the SVM scoring the cutoff was varied from −10 to 10, for the distance to the SS/Loop junction the cutoff was varied from 0 to 5 amino acids and for the conservation rank the cutoff was varied from 0 to 1. The points marked with circles are the maximum enrichment points of the true positive rate versus the false positive rate. The black dashed line indicates a random prediction.

These values are plotted in Figure 4 for each feature varying the cutoff. Figure 4 indicates that the distance to the active site, the SVM scoring and the location of insertion site to conserved sequences all show significant predictive power with areas under the curve (AUC) of 0.79, 0.76, and 0.73 respectively. The distance to loop-secondary structure junction did not show as significant predictive power, where there is a smaller enrichment in the true positive rate over the false positive rate (AUC of 0.54). We marked with circles the cutoff points that gave the best compromise of the of true positives rate (sensitivity) versus the false positive rate (1-selectivity), which is the furthest point from the diagonal. These are 14.1 Å for the distance to the active site or dimer interface site, 0 for the SVM score, 2 amino acids for the distance to the SS/Loop junction, and 0.61 for the conservation rank.

### Western Blotting of Intein Insertions in the XynB Xylanase

**Figure 5:**
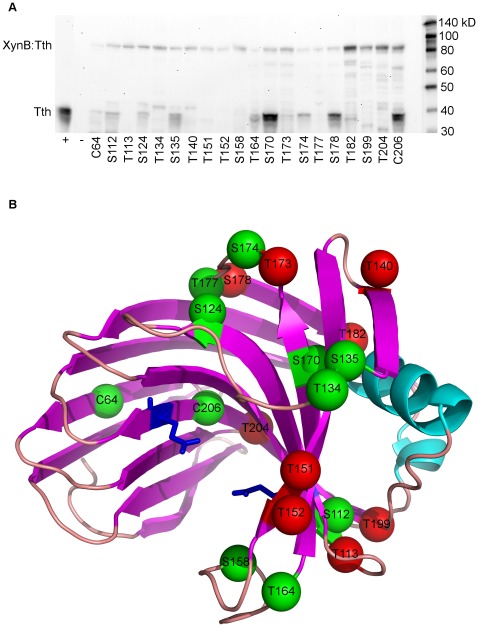
Western blot of Tth intein splicing at 20 sites of the XynB xylanase. A) The Tth intein was inserted into randomly selected 20 sites of the catalytic domain of the XynB generating XynB:Tth. E coli SOLR cell expressing the XynB and intein modified XynB:Tth were lysed, heat treated at 60°C/3 hours and Western blotted. Intein unmodified wild type enzyme XynB (+) and the empty vector control (–) are at the left lanes. Positions of the intein modified precursors (XynB:Tth) and the spliced product (XynB) are marked at the left. B) Insertion sites are mapped out on the crystal structure of XynB. Spice sites are shown as green spheres and non-splice sites are shown as red spheres.

**Figure 6:**
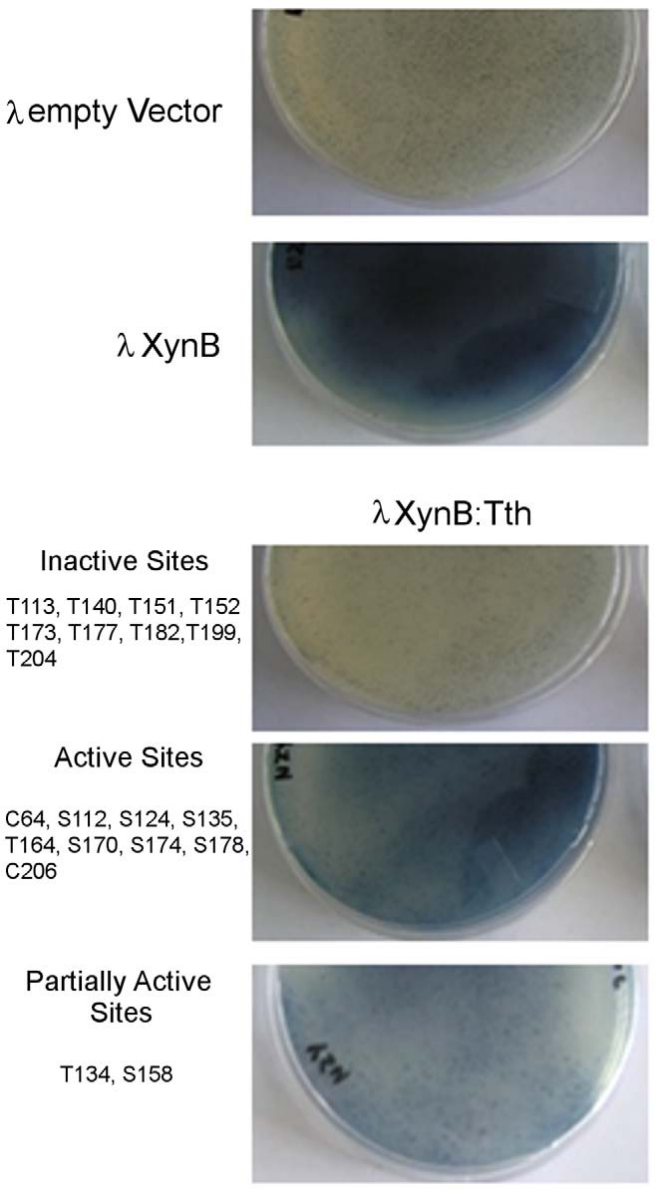
Xylanase diagnostic plate phenotypes of intein modified XynB (XynB:Tth) constructs. The T. thermophilus DnaE-1 intein (Tth) was inserted into 20 sites of XynB and expressed from lamda phage. λXynB:Tth infected E.coli were grown on xylanase diagnostic plates containing the insoluble substrate AZCL-xylane embedded in the top agarose. Xylanase activity was scored following heat treatment of the confluent lysis plates at 60°C/3 hr. Blue color development indicates xylanase activity. The plates shown are representative of the three different activity groups: active, partially active and inactive.

**Figure 7:**
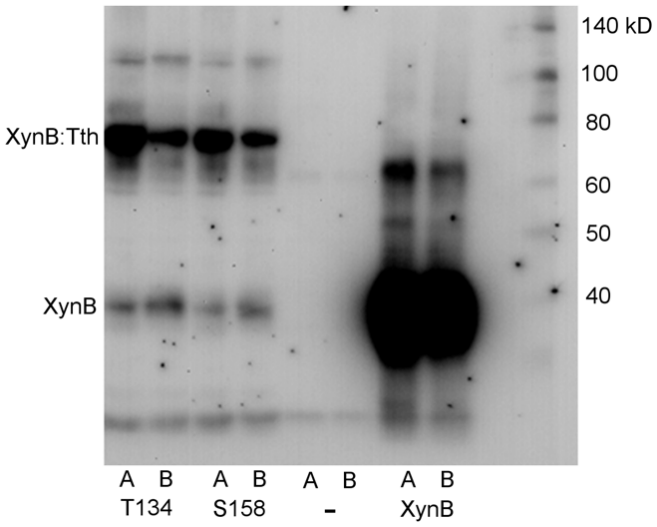
Western blot demonstrating intein splicing at the T134 and S158 sites of XynB. Intein mutagenized XynB:Tth clones were expressed from lamba phage. Phage infected E.coli were grown on xylanase diagnostic plates as in Fig.6, but at a lower plaque density that allowed scoring individual plaques for xylanase activity. Plaques that displayed distinctively strong blue color, indicative of high xylanase activity, were phagemid rescued to E.coli SOLR cells, and bacterium lysates expressing intein mutagenized XynB:Tth was Western blotted as in Figure 5. Duplicate samples (A and B) of two XynB:Tth clones with mutagenized intein inserted at the T134 and S158 sites are shown at the left. Empty vector control (−) and the intein unmodified XynB is at the right. The T134 and S158 site both support splicing.

We experimentally evaluated the suitability of 20 potential insertion sites in the XynB endo-1,4-beta xylanase from *Dictyoglomus thermophilum* (P77853) to facilitate splicing of the *Thermus thermophilus* DnaE-1 intein (Tth) based on these predictors. The tested sites included 20 different cysteine, serine, and threonine (C/S/T) sites between amino acid residues 64 to 206 of the catalytic domain. To evaluate the ability of insertion sites to support splicing we have Western blotted lysates of *E.coli* cells expressing XynB:Tth (Figure 5). In addition, we determined whether splicing of the intein from the XynB:Tth precursor can restore activity of XynB. We expressed the intein-modified protein XynB:Tth from lambda phage and scored enzyme activity in phage plaques on xylanase diagnostic plates (Figure 6). Western blots demonstrate splicing of the intein from the XynB:Tth precursor and accumulation of XynB at 10 of the 20 sites tested (Figure 5; sites C64, S112, S124, S135, T164, S170, T173, S174, S178 and C206). Intein splicing and XynB accumulation correlated with recovery of enzyme activity when comparing splicing profiles (Figure 5) with the enzyme activity on xylanase diagnostic plates (Figure 6); non-splicing precursors showed no enzyme activity, while splicing competent XynB::Tth precursors showed activity. There were three exceptions: insertion at T173 resulted in splicing but no activity, while insertions at sites T134 and S158 showed no detectable splicing but resulted in relatively low xylanase activity compared with the splicing competent XynB:Tth precursors (Figure 6). Splicing without recovery of activity at T173 indicates that intein insertion or intein splicing does not produce XynB in an active state. Activity without detectable splicing at T134 and S158 could indicate that the intein is partially tolerated and the precursors retain activity, or alternatively, T134 and S158 sites may support splicing but XynB is below the detection level of the immunoblot. However, inserting mutagenized Tth inteins into the T134 (P71L mutation) and S158 (R51G mutation) sites revealed both sites support splicing (Fig. 7). Therefore they were designated as splice sites.

### Evaluation of 20 Insertion Sites in the XynB Xylanase

Given the predictive power of the four features in identifying native-like intein insertion sites, we wanted to evaluate their utility in predicting intein insertion into a non-native host protein. To test their predictive capability, the native insertion site predictors were applied to the 20 insertion sites (Table 1) to see if they enriched the selection of sites that permitted splicing, given the optimal cutoff values (described previously for native inteins). If a predictor identifies all of these sites as permitting splicing, it would include the 11 positive splice sites and 9 non-splice sites. This would lie on top right of the diagonal that divides the ROC curve (Figure 4) thus giving no predictive power (TPR of 1 and FPR of 1). Methods that select a subset of these sites will show predictive power by having an increase in selectivity (decrease in FPR) that is larger than the decrease in the sensitivity (less decrease in TPR). This would be in top left half of a ROC curve. Looking at the SVM prediction, five positive splice sites have SVM scores of greater than 0 compared with two non-splicing sites. This gives a TPR of 45% (5 TP and 6 FN) and a FPR of 22% (2 FP and 7 TN). This increases the selectivity greatly with less loss in sensitivity. For the distance to the active site, seven sites are closer than 14.1 Å which were splicers, while six non-splice sites were closer. This gives a TPR of 64% (7 TP and 4 FN) and a FPR of 67% (6 FP and 3 TN). Here we lower the sensitivity and selectivity proportionally and show no predictive power. When looking at the distance to the loop-secondary structure junction, seven splice sites are within 2 residues of loop-secondary structure junction while five non-splice sites were within 2 amino acid residues. Here we have a TPR of 64% (TP and 4 FN) and a FPRof 55% (5 FP and 4 TN). This shows a slight improvement of predictive power as the TPR rate is greater than the FPR. Finally, seven of the splice sites fell above the 0.61 conservation rank, whereas only two non-splice sites did. This gives a TPR of 64% (7 TP and 4 FP) and a FPR 22% (2FP and 7 TN). This last method has the greatest increase in selectivity versus a loss in sensitivity and shows the most predictive power. The predictive power of these four methods is shown in a ROC curve in Figure S1. The usefulness of the predictors in this test set is somewhat consistent with earlier observations on the native insertion sites. The SVM score and the conservation rank predictors both show significant predictive power while the distance to the loop-secondary structure has only a slightly larger TRP than FPR so is less predictive. The exception to this is the distance to the active site residue which does not show the enrichment observed in native sites. If the three predictors that showed the most enrichment on native insertion sites are each required (conservation of sequence, SVM score and distance to the active site), three sites, S112, S158 and T164, are identified, all of which supported splicing.

**Table 1:** Analysis of XynB C/S/T sites for features seen in native insertion sites.

site	Distance toactive site (Å)	SVM score	Distance toSS junction†	Conservation rank	Splicing
C64	10.01	0.41	>2	0.55	X
S112	6.74	0.11	>2	0.85	X
T113	8.99	0.36	2	0.34	
S124	10.58	−1.34	2	0.68	X
T134	17.40	0.66	1	0.30	X
S135	17.22	−1.13	1	0.00	X
T140	21.71	−0.02	2	0.81	
T151	9.71	−0.29	>2	0.09	
T152	9.37	0.19	>2	0.43	
S158	8.15	0.34	2	0.91	X
T164	10.33	0.21	2	0.96	X
S170	12.06	−0.45	>2	1.00	X
T173	18.16	−1.47	1	0.49	
S174	17.45	−0.32	2	0.36	X
T177	13.89	−1.38	1	0.32	
S178	16.44	−0.01	1	0.62	X
T182	17.55	−0.15	>2	0.77	
T199	11.61	−0.60	1	0.23	
T204	11.54	−0.16	>2	0.51	
C206	6.43	−0.55	>2	0.70	X

† Distance to secondary structure-loop junction is the number of sites away in primary sequence.

### Structural Modeling of Intein Modified XynB

**Figure 8:**
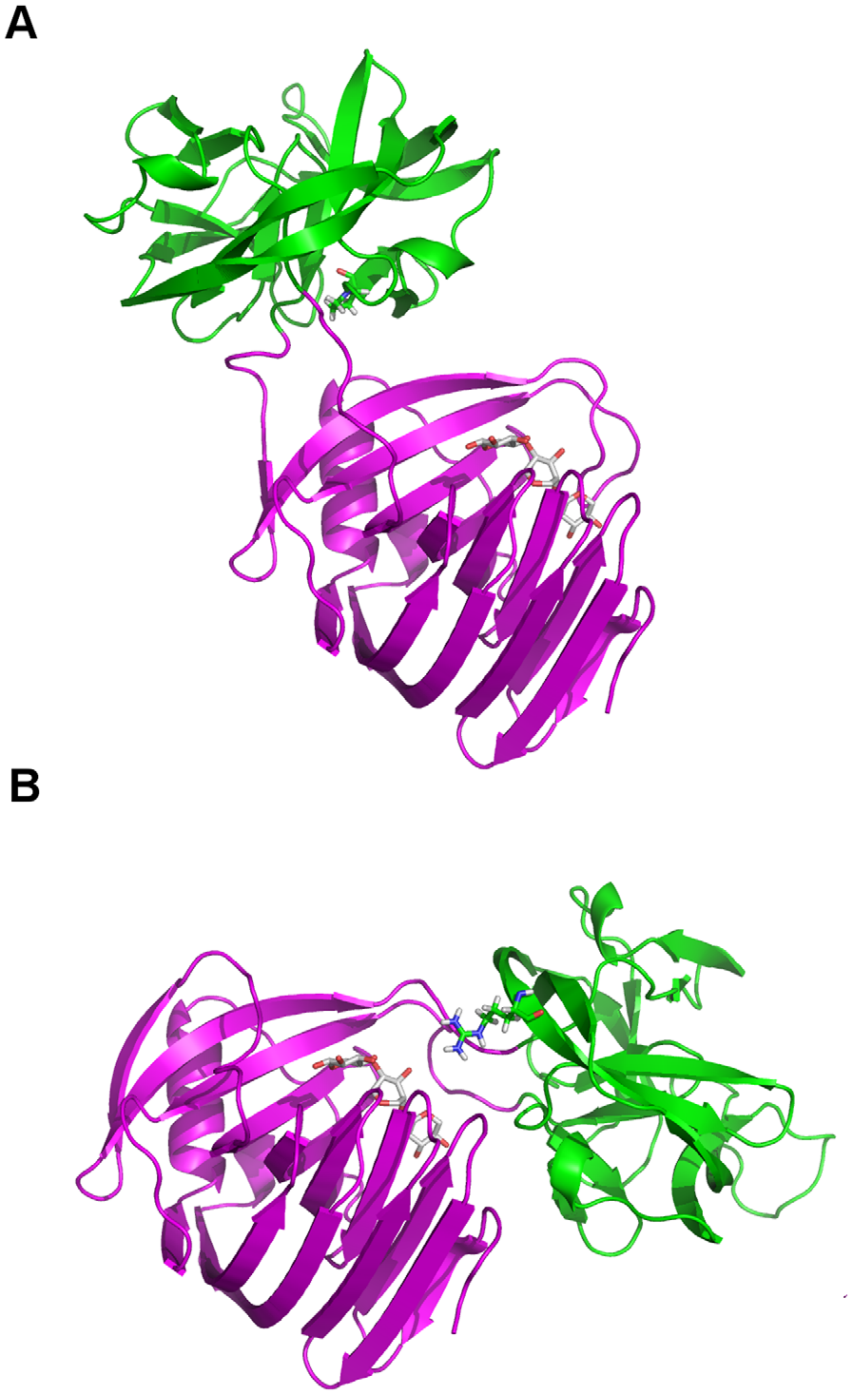
Structural models of the Tth-modified XynB enzyme. The Tth intein (green) is inserted into the catalytic domain of XynB (purple) at the (**a**) S158 site and the (**b**) T134 site. The substrate bound to the XynB binding pocket is shown in white sticks. The residues that were mutated in the native Tth sequence to allow for splicing are shown as sticks.

In the native insertion sites we saw a strong correlation between the insertion site and the distance to the active site residue. The fact that the intein is inserted close to the active site presumably correlates with the activity modulation associated with the native inteins insertions into their native hosts. However, this was not a strong predictor for insertion sites that support splicing in the XynB protein. Given that the ultimate goal is to create an intein based switch we wanted to attempt to understand the effect the location of the insertion site could have on intein splicing. For this, we constructed structural models of the intein modified XynB:Tth at two sites, S158 and T134. S158 is predicted by all four predictors, while T134 was predicted by the SVM model and distance to the SS/loop junction but not the distance to the active sites. The structural models show potentially significant differences between the two insertions in blocking of the active site. For the S158 insertion the intein is sitting directly on the opening to the binding pocket and may sterically hinder access (Figure 8a). In contrast the T134 insertion sits more distal to this pocket, only covering the side (Figure 8b). This difference was further investigated by the determining interaction of the intein with the XynB active site residues (E118 and E208). These two glutamate residues are relatively buried in the mature enzyme with solvent accessible surface areas of 11.5 and 10.8 Å^2^, respectively. For the T134 insertion these values are relatively stable, becoming 11.9 and 11.7 Å^2^, respectively. However, for the S158 insertion these are buried even more, decreasing to 0.0 and 8.6 Å^2^ respectively. This model suggests that the closer proximity of the S158 site allows for capping of the active site more completely which could translate into reduced activity in the intein-modified XynB. These differences suggest that the intein insertion might block activity simply by steric bulk as would any domain insertion and so the change in activity could just be coincidental to the intein insertion.

## Discussion

To support development of intein-modified enzymes, we evaluated features of cysteine, threonine, and serine sites in native exteins to determine whether any correlated with the identity of native insertion sites. Features we investigated that correlated to the location of the intein insertion included the conservation of the insertion site, similarity of the insertion site cassettes, local secondary structure, and distance to the active site and dimer interface. The distance to the active site/dimer interface, the conservation of the site and the SVM scoring all were shown to have strong predictive power in identifying native insertion site positions. Additionally, the distance to the secondary structure/loop interface and presence of β-sheet secondary structure were both enriched in the native insertion sites over the background of all C/S/T sites but were less predictive.

Our results agree with previous works that demonstrate intein insertion sites are commonly found in conserved regions of the protein [30], [31], [39]. The conserved insertion sites are thought to be important for continued functionality of the intein as precise cleavage is required for the host activity [4], to allow for horizontal transfer of the inteins into homologous hosts [31], or to provide an effective structural environment often seen in conserved sites [40]. We demonstrated in our test set of native intein insertion sites that this sequence conservation does strongly correlate with splicing. Additionally, in testing the insertion of Tth into XynB, sequence conservation of native intein insertion site also correlates strongly with splicing. This observation reinforces the analysis results and predictive value that inteins are concentrated in conserved sites because they are enriched with the appropriate structural and chemical environment that is conducive to splicing.

The similarity and the conservation amongst homologous host of the insertion site cassettes is an important property when it comes to insertion site prediction. Inteins are seen to function best with their own insertion site cassette, frequently requiring 2 to 6 flanking residues to be active [16], [41]. By generating the SVM model we could aid identification of non-native intein insertion site cassettes that are most similar to native insertion site cassettes.

Secondary structure features of the insertion site location were also shown to be significant for intein function. From a protein structure perspective, it is intuitive that the intein would require a location that has enough degrees of freedom to enable the structural changes associated with splicing [21], [22], [23]. Additionally, native domain insertions typically occur in regions connected by loops [39], and insertions into the middle of a well structured region could have negative effects on the folding of the host domain. By being close to a loop-secondary structure junction only a few residues need to change topology to accommodate the intein insertion. It is interesting that these sites do have some structural constraints. We found that the proportion of native insertion sites in the middle of loops is approximately the same as that of all C/S/T sites. This suggests that being close to a secondary structure/loop junction may be beneficial. This correlates with previous descriptions of insertion site preferences that find that insertions occur in active sites and binding regions because these tend to be located in loops between α-helices and β-sheets [40]. In addition to being close to a secondary structure-loop junction, the inteins are also biased towards β-sheet regions specifically. They are over 60% more likely to occur in this type of region than in all other C/S/T sites in the extein set examined. This bias complements the proposed mechanism of intein splicing which is thought to include interaction of extein residues with the intein to form a β-sheet topology [42], [43]. The propensity to form a β-sheet in this insertion site region could allow the intein to be more active.

The proximity of the insertion site to active site or dimer interface strongly correlates with the location of the insertion site. Previously, it had been suggested that active sites and dimer interface sites could be ideal locations for inteins because conserved sites that are not in the core tend to be active sites, binding and docking sites, and dimer interfaces with surface loop regions between α-helices and β-sheets [40]. However, if the proximity to the active site were mainly associated with the conservation of the site we would expect to see significant correlation between the conservation of an intein insertion site and its distance to the active site. This is not the case for our dataset of native exteins (R^2^ = 0.0154). The distance to the active site may be functionally important to the intein. By blocking activity in the precursor state, the host protein requires the intein to be functional for the host to retain its activity. This would create selective pressure on the intein to splice or the host protein itself will lose functionality. If the intein does not block the activity of the protein, then there is not the same pressure on the intein to be functional. The intein could be inactivated and the host protein could still function.

Our goal was to predict native like intein insertion sites in enzymes that do not natively host inteins and have these insertions permit intein splicing. Insertion site selection may be done by linker scanning or similar methods that can identify insertion tolerant sites [41]. However, many sites are not suitable to support splicing, thus the intein may benefit from being inserted with its flanking residues [16], [41] leaving a footprint upon intein splicing if these flanking residues are not native in the host protein. To maintain integrity of the host, an alternative approach relies on testing C/S/T sites for their ability to support splicing, which is often a labor intensive procedure. In the case study of the 20 C/S/T insertion sites of the XynB xylanase, we found that the native insertion site features (SVM score, conservation rank and distance to the closest catalytic residue) have predictive value in identifying splice sites or activity modulation, in particular when the three predictors are applied simultaneously.

The total computational time required for these predictive models is dominated by the generation of the homology model and the psi-blast search used to predict conservation rank. Each of these can takes up to 30 minutes on a single cpu depending on the size of the protein. After that the remaining calculations are done in seconds. This compares favorably to the 2 weeks required to perform the experimental validations of the 20 insertion sites of XynB. The predictive power we have seen from these methods can be used to prioritize the screening of a given protein, as well as to increase confidence in the selection of follow-up mutagenesis. As is the case for the T134 and S158 sites, mutagenesis to improve the splicing of an intein could take several weeks to months to complete. Any upfront pruning of possible sites could then save a lot of downstream experimental work by prioritizing the sites to focus on.

We have shown the predictive methods to be useful, but there is still room for improvement. This becomes apparent when comparing the sites predicted by to all the methods to be splicers (S112, S158 and T164 in Table 1) to the sites with the strongest splicing (S170, S178 and C206 in Figure 5). The latter are not predicted by all the methods to splice and none of these passes the SVM threshold. Looking at the possible problems with the prediction methods can help to guide us toward future improvements. One significant problem with the prediction methods could be associated with the construction of the native test, particularly in training the SVM model. The positive test set was constructed from a curated database of all known inteins, but only contained 400 examples of known inteins insertion site cassette. It is probable that the space of all suitable insertion sites is much larger than this and thus this set might limit the number of sequences that can be accurately predicted. Additionally, the negative test set was chosen based upon the assumption that all the rest of the sites most likely did not support splicing. Given the number of splice sites we see in the test case of XynB this may be a poor assumption. An increase in the number of experimentally verified positive and negative sites would greatly expand the predictive strength of all the methods. This can be done by systematically scanning a larger set of proteins as we have done for XynB.

Other shortcomings could be based on the reliance of homology models for the identification of structural properties with strong predictive power. The homology models were generated from template structures which had sequence similarity to the target intein with at least an E-value of less than 10^−10^. The accuracy of those models generated from templates with E-values close to 10^−10^ could be low and may not give atomic level accuracy of the entire protein. Additionally, given the large number of homology models generated we used a relatively simple method of structure prediction using a single fixed template with flexible loop prediction. Despite this it appears that these types of models are still useful when predicting fold level properties like secondary structure and approximate distances. However, we should have less confidence when these models are used to describe more atomistic properties like the flexibility of the sites or the degree of burial. Improvements to the models would give a more reliable assessment of the predictive power of these methods. This though requires isolating a larger set of insertion sites in proteins with known structures or using a more accurate but more computationally expensive structure prediction methods [44] on known exteins.

Structural modeling of the intein insertions at two sites that support splicing highlighted the role the location of the insertion site could play on the ability of the intein to suppress host enzyme activity, which is a desirable trait in developing regulated intein splicing for controlling host protein activity. These findings demonstrate the potential of insertion site specific features to enrich selection for sites capable to support intein splicing and the use of domain insertion models to get a better understanding of the role that the intein insertion will have on activity modulation. This method is much slower than the other prediction methods described in this paper and takes hundreds of hours of cpu-time to calculate the thousands of decoy models required. However, even this type of modeling can be used to analyze a few sites to provided an increased level of detail. This could aid in the prediction of sites that block activity, as well as, interacting intein and extein sites that can be the target of optimization through mutagenesis.

## Materials and Methods

### List of Inteins

Inteins for this set were selected from the NEB Inbase Intein Library [25], [26], [27]. This library contained 469 individual FASTA files as of July 2, 2009. This was filtered to eliminate all cases of trans-splicing inteins, bacterial intein-like domains (BILs), and other inteins whose precursor protein was not present in the NCBI database (http://www.ncbi.nlm.nih.gov/). The resulting set comprised of 412 inteins (see Supporting Materials). From this set of inteins, the corresponding precursor proteins were extracted from the NCBI genpept library. This was done using the GI accession number from the Inbase library as well as using a Blast search to identify cases where the accession number was missing or not up-to-date. The intein insertion site was then confirmed, the intein was removed from this precursor protein and the native extein was defined as the remainder of the sequence.

### Conservation of Intein Insertion Sites

Conservation of the intein insertion site was determined using an entropy based position-specific scoring matrix (PSSM) based method. The PSSM was calculated using the NCBI PsiBlast program [45]. For each sequence the stand alone program blastpgp from the blast suite version 2.2.18 was run using the default parameters with the use of composition-based statistics set to true, filter query sequences set to false, maximum passes set for the psiblast to 2, number of one-line descriptions set to 10000, number of alignments to show set to 10000, number of best hits to a region to keep set to 1000, the E-value threshold to keep for the PSSM set to 10^−15^, and the expectation value set to 10^−15^.

From the PSSM for each sequence, the sequence entropy (S_i_) for each site *i* was calculated using equation 3, where p_ij_ is the probability of amino acid type *j* at site *i*.
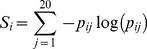
(3)


### Similarity of Intein Insertion Site Cassettes Using a Support Vector Machine (SVM)

To generate a SVM of the intein insertion site cassettes, a subset of inteins was chosen to minimize the bias of sequence homology. This was done by removing sequences from the dataset that had identical insertion site cassettes (defined as the −3 to −1 and the +2 to +4 positions). When two sequences had identical cassettes, the sequence with the larger extein was kept. This reduced the total to 188 extein cassettes. Using this set of 188 native exteins, a SVM model was generated to predict the intein insertion site. The training set was determined by using the native intein insertion site as a true positive, and 3 other random C, S or T cassettes as true negative sites. For each insertion site *i,* a 120-dimensional vector (**v_i_**) was generated, which describes the cassette surrounding the site. This vector is a concatenation of six 20-dimensional vectors (**s**_p_), one for each of position of the cassette (−3 to −1 and +2 to +4). These are represented in equation 4.
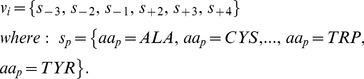
(4)


Here for each vector **s**_p_, all 20 components are 0 except for the component corresponding to the amino acid present at that site. This has a value of 1. To train the SVM model the positive set were given a target value of +1. In addition, three true negatives were chosen from each extein as a random T, S or C site in the extein (the three additional cassettes did not include any duplicates). These were given a target value of −1. The SVM model was then trained using SVMlight version 6.02 [33], [34] with a linear kernel and a cost-factor of 3 that weighed errors in the positive value 3 times over errors in the negative values. This was done since there are 3 times as many true negative cassettes.

This method was tested using a leave-one-out cross validation. For each target extein, the remaining 187 exteins were selected as the training set using the same training method as for the whole set. All C, S, and T sites were then scored for the target extein using the SVM and the rank of the true positive was determined as the fraction of decoy insertion site cassettes with a score lower than the true positive insertion site. This was repeated 25 times for each extein using a different random training set.

### Homology Models of Exteins

All 412 exteins were blasted against the sequences contained in the PDB (4/20/2010). Any extein sequence that had a hit with an E-value of 10^−10^ or lower was included in this set to generate homology models. This included 300 extein sequences. These sequences and the alignments to the blast hits were then used to generate homology models. The top blast hit was downloaded from the PDB and all non-aligning structure was removed. The sequence of the extein was then repacked onto the template structure using Rosetta v3.1 fixed backbone repacking. All non-identical residues were repacked and residues that were identical in the alignment were held fixed. Ten different repacks were performed and the lowest energy one was selected. Parts of the alignments with gaps were then predicted using the loop modeling module of Rosetta v3.1 loop model with the ccd_closure method. This set of structures was then filtered to limit it to non-identical sequences. When structures had identical sequences, the structure with the longer sequence was chosen.

### Secondary Structure Assignment

Using the homology models constructed for all of the exteins, the local secondary structure for all possible insertion sites were determined and compared to the native secondary structures. Their Dictionary of Protein Secondary Structure (dssp) secondary structure assignments were made using the program Stride with default parameters. [36] These were further re-categorized by classifying types G, I and B as “other”, and T, S and C as “loops”. Using these class designations, all of the intein insertion sites were classified into six local structure types of: bordering (being within 2 amino acids of) a beta-sheet-loop junction, bordering an alpha-helix-loop junction, in a beta-sheet, in an alpha-helix, in a loop (more than 2 amino acids from the end), or part of some other structural motif.

### Flexibility of Intein Insertion Sites

The flexibility of insertion sites were determined using molecular dynamics equilibration simulations. The initial structures used were from the set of homology models described above. These structures were solvated into a solvent sphere of TIP3 waters using the VMD solvate package. [46] The solvent sphere radius was set to 5 Å larger than the largest Cartesian distance of any atom of the protein from the center of mass of the protein. These solvated proteins were then minimized for 1000 steps followed by a 5 ns molecular dynamics simulation using the program namd2. [47] For these simulations the Charmm27 parameter set was used, the forcefield parameters used the exclude scaled 1–4 value with a scaling of 1–4 interactions set to 1. The cutoff for interactions was set to 12 Å with switching set to on and the start of the switch distance set to 10. Additionally the limit of the pair list distance was set to 13.5 Å. The time step for the equilibration was set to 2 fs/step with all bonds set to be rigid. Additionally, the Langevin dynamics setting was turned on with a damping coefficient set to 5/ps. Snap shots of the simulation trajectories was taken every 25000 steps. For the collection phase the 50 snapshots from the last 2.5 ns of the simulation were then used to calculate the flexibility of all C/S/T sites in the enzyme. This was done by calculating the RMSD of the Cα atoms at these sites as compared to the first snapshot in the collection phase. The protein structure for each snapshot was structurally aligned to the first snapshot. For a given residue *i* the rmsd was defined as in equation 5, where x_i,s_ is the position of the Cα-atom of residue *i* at in snapshot s.
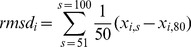
(5)


### Degree of Burial

The degree of burial of the C/S/T sites was determined by calculating the C_β_ density from the homology model described above. This calculation was performed by counting that number of C_β_ atoms that are within 8 Å of the target site C_β_ atom. The ranks for the cysteine, serine and threonine residues were compared separately for the native and non-native intein insertion sites.

### Proximity to Active Site and Dimer Interface

To determine the distance of the intein splice site to the active site of the protein or dimer interface, information for the exteins was extracted from the NCBI genpept database. To find the active site positions the sequences of the extein were blasted against the NCBI nr sequence database using PsiBlast [45] (with an inclusion criteria of Eval = 0.00005). The top 500 sequences were taken from the PsiBlast and checked to determine if they had sites with *type = “active site”* described in their “Features” section of their genpept file. All active site residues identified whose alignment was to part of the extein sequence in the homology model and had identical residue types were set as the active site residues. The sequence alignment that produced active site residues with the lowest E-value was used. All PsiBlast alignments gave E-values of 10^−100^ or lower. For all homology models in which there were both an intein insertion site and an active site residue contained in the protein, the distance between the C_α_ atoms of these positions was calculated. The closest distance for any active site to the insertion site was set as the distance for that insertion site position. A similar method was used to find the dimer interface positions. The only difference was that the list of dimer interface sites was determined by identifying sites that had type = “other” and note = “dimer interface”. The distances were calculated as above described above.

### Intein Insertion into XynB, Phage Expression and Activity Testing on Xylanase Diagnostic Plates

XynB xylanase from *D. thermophilum* (Uniprot accession # P77853) [48], [49] was corn codon optimized and 6×His tagged at the C-terminus. The *T. thermophilus* DnaE-1 intein (Tth) was corn codon optimized, inserted 5′to 20 C/S/T sites of XynB (C64, S112, T113, S124, T134, S135, T140, T151, T152, S158, T164, S170, T173, S174, T177, S178, T182, T199, T204 and C206) using overlapping PCR and cloned into the EcoRI and XhoI site of the precut λ Uni-ZAP XR expression vector (Agilent Technologies). Packaging, phage infection and plating was done as described in the lambda ZAP-II manual (Stratagene), except that the top agar of the diagnostic plate was supplemented with IPTG (2.5 mM) and 0.2% AZCL-xylane oat (Megazyme) to detect xylanase activity.

To score xylanase activity of the intein modified XynB:Tth, plates were incubated at 37°C overnight till confluent lysis then incubated at 60°C for 3 hrs. Blue color development indicated xylanase activity.

### Western Blot

XynB:Tth and the control XynB were phagemid rescued from λ UniZapII to SOLR according to the manufacturer’s protocol (Stratagene) and were grown in 1 ml auto induction medium (Novagen) supplemented with Carbenicillin (100 mg/l) at 37°C overnight. Cells were pelleted, resuspended in 100 µl of polybuffer pH = 6.5 containing 1×FastBreak Lysis Buffer™ (Promega) and 1/5000 dilution of Benzonase (Novagen) and lysed at 30°C for 1 hr. Lysate was diluted into 400 µl Polybuffer pH = 6.5 and an aliquot was heated at 60°C for 3 hrs. 2.5 ul of lysate was separated on a 12% SDS/PAGE (BioRad), transferred onto PVDF membrane and the Western blot was developed using the “THE mouse anti-6×His” (Genscript, Piscataway, NJ) primary antibody at 0.25 µg/ml, and the secondary antibodies peroxidase-goat anti-mouse IgG (A4416, Sigma, St. Louis, MO; 1∶4000 dilution) and the HRP-anti-biotin (Cell Signalling; 1∶5,000 dilution) to detect the biotinylated mol. weight markers (Cell Signalling).

To test the intein mutagenized XynB:Tth at the T134 and S158 insertion site, Tth intein was random mutagenized using Mutazyme (Stratagene), inserted into XynB and expressed from the lambda ZAP-II onto xylanase diagnostic plates as above, but the phage titer for plating was reduced to grow individual plaques. Plaques that developed distinctively strong blue color indicative of high xylanase activity were phagemid rescued to SOLR and lysates of bacteria expressing the intein mutagenized XynB::Tth were Western blotted as above.

### Models of Intein Modified Enzymes

Three dimensional models of the Tth DnaE-1 intein (Tth) inserted into the S158 and T134 sites of the enzyme XynB from endo-1,4-beta xylanase from *Dictyoglomus thermophilum* (XynB) were generated by inserting the homology model of the Tth intein into the S158 and T134 sites of a X-ray crystal structure of the XynB catalytic domain (pdbID 1f5j). Chain A of 1f5j was used and renumbered to correspond to XynB full length sequence (Uniprot ID P77853). Thus residue 1 in the structure was renumbered to be residue 29. This was done using the domain insertion module of Rosetta++ v2.3 (1). The intein Tth homology model was generated using SwissModel [50], [51], [52] and the Tth intein sequence from GenPept (gi: 46200108, residues 768–1190). Briefly, this sequence was aligned against sequences from the Protein Databank NCBI sequence database using NCBI Blast blastp with defaults parameters. [45], [53] This resulted in two separate hits from the start and end of the sequence to the pdb structure 2IMZ chain A and B. Because there was not a significant hit to the endonuclease domain the sequence was trimmed to approximate the removal of the endonuclease domain (amino acids 1–102, 379–423), and aligned against the PDB sequence database. This resulted in higher scoring alignments of the N- and C-terminal domains of the RecA mini-intein with the Tth intein sequence. Using this sequence alignment a homology model of Tth (without the endonuclease domain) was constructed using Swiss Model.

With the structure of XynB and Tth, structural models of the XynB-T134 and -S158 Tth intein modified enzymes were generated. To generate these models we aligned the two domains such that the intein insertion site of the extein was close to and facing the N- and C- termini of the intein. This was done by first identifying the Cα atoms of the +1 position (extC) and the −1 position extN and the N-terminal (intN) and the C-terminal intein position (intC). The intein and the extein were aligned to minimize the extC-intC and the extN-intN distances as well as maximize (closest to 180°) the angle formed by the center of mass of the intein, the midpoint (MP) of the positions extC, intC, extN and intN and the center of mass of the extein. The intein was then moved away along the axis formed by the center of mass of the intein and MP in 0.5 Å increments until no backbone atom clashes occurred between the intein and the extein. For each insertion site, domain insertion were performed using the program and protocol as described in Berrondo et al. [54] with a few exceptions. Briefly, 1625 initial conformations were generated by random translating and rotating the intein about the center of the points intC-intN-extC-extN. The rotations were made in a range of +/−0.5 radians. The translations were +/−2 Å. From each starting structures 5 separate low-resolution models were generated using the domain insertion module Rosetta++ v2.3 and default parameters and without an initial randomization step. Of the resultant 8125 structures, those that had a near-native-like intein domain (<2 Å rmsd, and less than 15 Å distance between the intC and intN C_α_ atoms) were refined using the high-resolution refine module. The lowest energy model for each the T134 and S158 insertion site was chosen as the best approximate model.

## Supporting Information

Figure S1 **ROC curve of the prediction of Tth intein insertion sites in XynB.** The true-positive rate and the false-positive rate was calculated for the 4 features used to predict the Tth intein insertion into 20 different sites of XynB: (blue) distance to the active site or dimer interface, (green) SVM score, (red) distance to SS/Loop junction and (cyan) sequence conservation. These rates were determined over a range of cutoff values which were: for the distance to the active site or dimer interface site the cutoff value was varied from 0 to 30 Å, for the SVM scoring the cutoff was varied from −10 to 10, for the distance to the SS/Loop junction the cutoff was varied from 0 to 5 amino acids and for the conservation rank the cutoff was varied from 0 to 1. The points marked with circles are the maximum enrichment points of the true positive rate versus the false positive rate for the native intein insertion sites. The black dashed line indicates a random prediction.
(TIF)

Table S1 **List of inteins, exteins, insertion sites and insertion site cassettes.** All sequence numbering is based upon the full length precursor protein.
(DOC)
