# Supplementary figures and images for: A Predictive Model of Intein Insertion Site for Use in the Engineering of Molecular Switches
Source: PLoS One. 2012 May 23;7(5):e37355. doi: 10.1371/journal.pone.0037355 (PMC3359363; doi:10.1371/journal.pone.0037355)

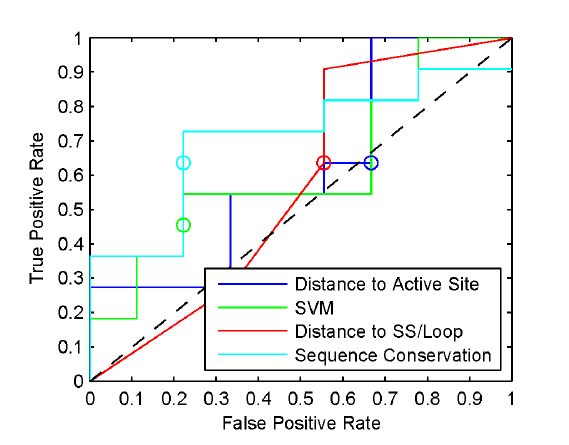

Supplement: Figure S1 — ROC curve of the prediction of Tth intein insertion sites in XynB. The true-positive rate and the false-positive rate was calculated for the 4 features used to predict the Tth intein insertion into 20 different sites of XynB: (blue) distance to the active site or dimer interface, (green) SVM score, (red) distance to SS/Loop junction and (cyan) sequence conservation. These rates were determined over a range of cutoff values which were: for the distance to the active site or dimer interface site the cutoff value was varied from 0 to 30 Å, for the SVM scoring the cutoff was varied from −10 to 10, for the distance to the SS/Loop junction the cutoff was varied from 0 to 5 amino acids and for the conservation rank the cutoff was varied from 0 to 1. The points marked with circles are the maximum enrichment points of the true positive rate versus the false positive rate for the native intein insertion sites. The black dashed line indicates a random prediction. (TIF) [file pone.0037355.s001.tif]
