# Supplementary material for: A Predictive Model of Intein Insertion Site for Use in the Engineering of Molecular Switches
Source: PLoS One. 2012 May 23;7(5):e37355. doi: 10.1371/journal.pone.0037355 (PMC3359363; doi:10.1371/journal.pone.0037355)
Supplement: Table S1 — List of inteins, exteins, insertion sites and insertion site cassettes. All sequence numbering is based upon the full length precursor protein. (DOC) [file pone.0037355.s002.doc]

| Intein | GenPept ID | Extein | Intein | Insertion Site | Cassette Sequence |
| --- | --- | --- | --- | --- | --- |
| Abr PRP8 | 61191911 | 1-54 220-227 | 55-219 | S220 | ERASGFE |
| Afu-Af293 PRP8 | 70989177 | 1-1522 2342-3170 | 1523-2341 | S2342 | ERASGFE |
| Afu-FRR0163 PRP8 | 56418438 | 1-14 834-838 | 15-833 | S834 | ERASGFE |
| Afu-NRRL5109 PRP8 | 83274377 | 1-4 824-827 | 5-823 | S824 | ERASGFE |
| Agi-NRRL6136 PRP8 | 83274385 | 1-55 223-232 | 56-222 | S223 | ERASGFE |
| Ani-FGSCA4 PRP8 | 49094398 | 1-1511 2117-2945 | 1512-2116 | S2117 | EKASGFE |
| APMV Pol | 55416941 | 1-1052 1404-1740 | 1053-1403 | T1404 | YGDTDSI |
| Avi PRP8 | 61191913 | 1-54 224-231 | 55-223 | S224 | ERASGFE |
| Bci PRP8 | 154310560 | 1-258 1070-1899 | 259-1069 | S1070 | EKASGFE |
| Bde-JEL197 RPB2 | 83415453 | 1-120 609-1202 | 121-608 | C609 | GMVCPAE |
| Ceu ClpP | 1168975 | 1-447 904-1010 | 448-903 | S904 | QPESSIQ |
| Cga PRP8 | 37695593 | 1-32 203-219 | 33-202 | S203 | EKASGFE |
| Cgl VMA | 29420853 | 1-276 692-992 | 277-691 | C692 | YVGCGER |
| CIV RIR1 | 7461167 | 1-271 611-959 | 272-610 | C611 | SNLCSEI |
| Cla PRP8 | 61105788 | 1-59 582-596 | 60-581 | S582 | EKSSGFE |
| Cmo ClpP | 156619231 | 1-447 904-1010 | 448-903 | S904 | QPESSIQ |
| Cmo RPB2 (RpoBb) | 156619305 | 1-867 1563-1661 | 868-1562 | S1563 | KMHSRST |
| Cne-A PRP8 (Fne-A PRP8) | 37695591 | 1-34 206-215 | 35-205 | S206 | EKASGFE |
| Cne-AD PRP8 (Fne-AD PRP8) | 18028924 | 1-47 220-236 | 48-219 | S220 | EKASGFE |
| Cne-JEC21 PRP8 | 57222732 | 1-1530 1703-2532 | 1531-1702 | S1703 | EKASGFE |
| Cre RPB2 | 68300857 | 1-1085 1517-1607 | 1086-1516 | C1517 | ERDCIIS |
| Cst RPB2 | 83415447 | 1-692 1055-1080 | 693-1054 | S1055 | KIHSRSR |
| Ctr ThrRS | 255732025 | 1-399 745-1059 | 400-744 | C745 | PMNCPGH |
| Ctr VMA | 1076955 | 1-283 755-1088 | 284-754 | C755 | YVGCGER |
| CV-NY2A ORF212392 | 157952812 | 1-438 830-1612 | 439-829 | T830 | SGKTVSA |
| CV-NY2A RIR1 | 157953136 | 1-436 773-1103 | 437-772 | C773 | SNLCNEI |
| CZIV RIR1 | 62996800 | 1-161 301-378 | 162-300 | C301 | SNLCSEI |
| Ddi RPC2 | 66806019 | 1-505 970-1608 | 506-969 | C970 | GEACGLV |
| Dhan GLT1 | 58761325 | 1-1183 1791-2738 | 1184-1790 | C1791 | MRRCHLN |
| Dhan VMA | 49654817 | 1-271 666-999 | 272-665 | C666 | YIGCGER |
| Eni PRP8 | 62632793 | 1-9 615-618 | 10-614 | S615 | EKASGFE |
| Eni-FGSCA4 PRP8 | 105751883 | 1-1525 2131-2959 | 1526-2130 | S2131 | EKASGFE |
| Fte RPB2 (RpoB) | 156619181 | 1-95 473-584 | 96-472 | S473 | KMHSRSV |
| Gth DnaB | 6014983 | 1-376 537-599 | 377-536 | S537 | ESGSIEQ |
| HaV01 Pol | 70568331 | 1-646 879-1144 | 647-878 | T879 | YGDTDSV |
| IIV6 RIR1 | 15078798 | 1-271 611-959 | 272-610 | C611 | SNLCSEI |
| Kex-CBS379 VMA | 27528478 | 1-18 521-593 | 19-520 | C521 | YVGCGER |
| Kla-CBS683 VMA | 27526577 | 1-18 429-501 | 19-428 | C429 | YVGCGER |
| Kla-IFO1267 VMA | 29420855 | 1-276 687-978 | 277-686 | C687 | YVGCGER |
| Kla-NRRLY1140 VMA | 49644461 | 1-283 694-1027 | 284-693 | C694 | YVGCGER |
| Lel VMA | 149245851 | 1-279 701-1034 | 280-700 | C701 | YVGCGER |
| Nau PRP8 | 83274379 | 1-55 220-229 | 56-219 | S220 | ERASGFE |
| Nfe-NRRL5534 PRP8 | 83274383 | 1-43 199-208 | 44-198 | S199 | ERASGFE |
| Nfi PRP8 | 61191907 | 1-14 532-539 | 15-531 | S532 | ERASGFE |
| Ngl-FR2163 PRP8 | 61191901 | 1-54 210-217 | 55-209 | S210 | ERASGFE |
| Ngl-FRR1833 PRP8 | 61191903 | 1-54 224-231 | 55-223 | S224 | ERASGFE |
| Nqu PRP8 | 83274381 | 1-38 208-215 | 39-207 | S208 | ERASGFE |
| Nspi PRP8 | 61191899 | 1-54 224-231 | 55-223 | S224 | ERASGFE |
| Pbr PRP8 | 226292897 | 1-1524 2098-2926 | 1525-2097 | S2098 | ERASGFE |
| Pch PRP8 | 94442879 | 1-50 208-237 | 51-207 | S208 | EKASGFE |
| Pex PRP8 | 94442883 | 1-50 213-242 | 51-212 | S213 | EKASGFE |
| Pgu GLT1 | 146415518 | 1-1186 1740-2679 | 1187-1739 | C1740 | MRKCHLN |
| Pgu-alt GLT1 | 190347984 | 1-1186 1740-2679 | 1187-1739 | C1740 | MRKCHLN |
| Pno RPA2 | 169600505 | 1-710 1127-1616 | 711-1126 | C1127 | MYQCQMG |
| Ppu DnaB | 1706463 | 1-361 512-568 | 362-511 | S512 | ESGSIEQ |
| Pst VMA | 126094817 | 1-282 732-1065 | 283-731 | C732 | YVGCGER |
| Pvu PRP8 | 94442881 | 1-50 212-241 | 51-211 | S212 | EKASGFE |
| Pye DnaB | 90819089 | 1-361 510-566 | 362-509 | S510 | ESGSIEQ |
| Sas RPB2 | 83415495 | 1-697 1052-1058 | 698-1051 | S1052 | KIHSRAR |
| Sca-CBS4309 VMA | 27528482 | 1-18 536-608 | 19-535 | C536 | YVGCGER |
| Sca-IFO1992 VMA | 29420857 | 1-265 783-1101 | 266-782 | C783 | YVGCGER |
| Scar VMA | 27528472 | 1-18 473-545 | 19-472 | C473 | YVGCGER |
| Sce VMA | 67951 | 1-283 738-1071 | 284-737 | C738 | YVGCGER |
| Sce-DH1-1A VMA | 16417188 | 1-28 483-516 | 29-482 | C483 | YVGCGER |
| Sce-OUT7091 VMA | 29420841 | 1-258 713-1022 | 259-712 | C713 | YVGCGER |
| Sce-OUT7112 VMA | 29420843 | 1-258 713-1022 | 259-712 | C713 | YVGCGER |
| Sce-YJM789 VMA | 151941820 | 1-283 844-1177 | 284-843 | C844 | YVGCGER |
| Sda VMA | 27528474 | 1-18 520-592 | 19-519 | C520 | YVGCGER |
| Sex-IFO1128 VMA | 29420861 | 1-267 767-1067 | 268-766 | C767 | YVGCGER |
| She RPB2 (RpoB) | 115350032 | 1-578 969-1080 | 579-968 | S969 | KIHSRSS |
| Sja VMA | 213403900 | 1-277 754-1088 | 278-753 | C754 | YVGCGER |
| Spa VMA | 29420869 | 1-269 724-1046 | 270-723 | C724 | YVGCGER |
| Sun VMA | 27528480 | 1-18 433-505 | 19-432 | C433 | YVGCGER |
| Tgl VMA | 27528476 | 1-18 475-547 | 19-474 | C475 | YVGCGER |
| Tpr VMA | 27529083 | 1-18 474-546 | 19-473 | C474 | YVGCGER |
| Ure PRP8 | 258576163 | 1-1509 1690-2505 | 1510-1689 | S1690 | ERASGFE |
| Vpo VMA | 27526579 | 1-18 452-519 | 19-451 | C452 | YVGCGER |
| WIV RIR1 | 62996798 | 1-173 312-330 | 174-311 | C312 | SNLCSEI |
| Zba VMA | 27529077 | 1-18 475-547 | 19-474 | C475 | YVGCGER |
| Zbi VMA | 27529079 | 1-10 461-533 | 11-460 | C461 | YVGCGER |
| Zro VMA | 27529081 | 1-18 469-541 | 19-468 | C469 | YVGCGER |
| Aae RIR2 | 7521621 | 1-229 576-696 | 230-575 | C576 | DELCHVT |
| Aave Hyp-1721 | 120588868 | 1-2211 2545-2857 | 2212-2544 | T2545 | ALFTFKT |
| Aave RIR | 120587830 | 1-301 690-1226 | 302-689 | C690 | TNPCGEQ |
| Aba Hyp-02185 | 183210099 | 1-60 210-663 | 61-209 | T210 | GHGTGKT |
| Ace RIR1 | 117928683 | 1-156 310-1098 | 157-309 | C310 | VSACFIL |
| Aeh DnaB-1 | 78701429 | 1-226 728-1381 | 227-727 | T728 | MGKTTWA |
| Aeh DnaB-2 | 78701429 | 1-892 1311-1381 | 893-1310 | S1311 | LRESGAI |
| Aeh RIR1 | 78522210 | 1-325 703-1095 | 326-702 | C703 | TNPCGEQ |
| Ama MADE823 | 196111421 | 1-144 489-913 | 145-488 | S489 | HGKSELA |
| AP-APSE1 dpol | 6118040 | 1-608 915-993 | 609-914 | C915 | GKSCENI |
| Arsp-FB24 DnaB | 116669570 | 1-51 384-397 | 52-383 | T384 | ASETRQL |
| Bce-MCO3 DnaB | 170733229 | 1-226 574-807 | 227-573 | T574 | MGKTAFS |
| Bce-PC184 DnaB | 84354845 | 1-226 574-807 | 227-573 | T574 | MGKTAFS |
| BsuP-M1918 RIR1 | 15211863 | 1-30 416-474 | 31-415 | C416 | SNLCSEV |
| BsuP-SPBc2 RIR1 | 9630286 | 1-380 766-1084 | 381-765 | C766 | SNLCSEV |
| Bvi IcmO | 134132609 | 1-200 343-855 | 201-342 | C343 | LGFCITF |
| Cag RIR1 | 78188679 | 1-453 824-1513 | 454-823 | C824 | SNPCSEY |
| Cau SpoVR | 222524334 | 1-278 555-774 | 279-554 | S555 | GWASYWH |
| CbP-C-St RNR | 78675352 | 1-333 664-978 | 334-663 | C664 | ASCCRLT |
| CbP-D RNR | 83280998 | 1-333 664-978 | 334-663 | C664 | ASCCRLT |
| Cbu-Dugway DnaB | 29654195 | 1-389 536-610 | 390-535 | S536 | LRESGAI |
| Cbu-Goat DnaB | 120575469 | 1-389 536-610 | 390-535 | S536 | LRESGAI |
| Cbu-RSA334 DnaB | 165917759 | 1-389 536-610 | 390-535 | S536 | LRESGAI |
| Cbu-RSA493 DnaB | 154707365 | 1-389 536-610 | 390-535 | S536 | LRESGAI |
| Cce Hyp1-Csp | 171699128 | 1-319 728-801 | 320-727 | C728 | YCSCSHG |
| Cch RIR1 | 78170878 | 1-453 824-1513 | 454-823 | C824 | SNPCSEY |
| Ccy Hyp1-Csp-1 | 126617907 | 1-77 272-996 | 78-271 | C272 | DIGCGMA |
| Ccy Hyp1-Csp-2 | 126617907 | 1-513 923-996 | 514-922 | C923 | YCSCSHG |
| Chy RIR1 | 78044854 | 1-270 616-1101 | 271-615 | C616 | TNPCGEQ |
| Ckl PTerm | 146337057 | 1-146 549-993 | 147-548 | T549 | NGKTTLI |
| CP-P1201 Thy1 | 157310928 | 1-88 434-607 | 89-433 | S434 | KHHSVLE |
| Cth TerA | 48860529 | 1-67 401-849 | 68-400 | S401 | NGKSELA |
| Cwa DnaB | 45528273 | 1-213 710-941 | 214-709 | T710 | MGKTAFG |
| Cwa PEP | 46118766 | 1-453 848-1222 | 454-847 | C848 | GRTCHAA |
| Cwa RIR1 | 46120272 | 1-462 810-1115 | 463-809 | C810 | VNLCTES |
| Daud RIR1 | 169831430 | 1-268 644-1121 | 269-643 | C644 | TNPCGEQ |
| Dge DnaB | 94986136 | 1-215 617-849 | 216-616 | T617 | MGKTAFA |
| Dha-DCB2 RIR1 | 23114639 | 1-241 608-1111 | 242-607 | C608 | TNPCGEQ |
| Dha-Y51 RIR1 | 89893068 | 1-283 650-1153 | 284-649 | C650 | TNPCGEQ |
| Dra RIR1 | 15807364 | 1-524 891-1418 | 525-890 | C891 | TNPCGEI |
| Dth UDP GD | 206740307 | 1-261 743-917 | 262-742 | C743 | GGHCIPI |
| Dvul ParB | 120601920 | 1-286 575-684 | 287-574 | C575 | HEPCWYA |
| EP-Min27 Primase | 163955733 | 1-371 714-860 | 372-713 | T714 | HGKTEVV |
| Fal DnaB | 111226180 | 1-256 665-890 | 257-664 | S665 | VGKSTLG |
| Fsp-CcI3 RIR1 | 86742189 | 1-155 450-1281 | 156-449 | C450 | VSACFIL |
| Gob DnaE | 168700398 | 1-761 1208-1617 | 762-1207 | S1208 | FNKSHTA |
| Gob Hyp | 168697888 | 1-286 574-681 | 287-573 | C574 | HEPCLYG |
| Gvi DnaB | 35210594 | 1-221 480-709 | 222-479 | T480 | MGKTAFS |
| Gvi RIR1-1 | 37523535 | 1-193 607-1566 | 194-606 | S607 | GNKSRRG |
| Gvi RIR1-2 | 37523535 | 1-755 1123-1566 | 756-1122 | C1123 | TNPCGEI |
| Hhal DnaB | 121588855 | 1-225 718-1098 | 226-717 | T718 | MGKTTVA |
| Kra DnaB | 67985138 | 1-416 845-903 | 417-844 | S845 | ESGSIEQ |
| LLP-KSY1 PolA | 108861440 | 1-409 713-895 | 410-712 | C713 | AKACNFS |
| LP-phiHSIC Helicase | 58220027 | 1-34 374-953 | 35-373 | C374 | ATGCGKS |
| Maer-NIES843 DnaB | 166087682 | 1-379 522-585 | 380-521 | S522 | ESGSIEQ |
| Mav DnaB | 12958160 | 1-232 570-795 | 233-569 | S570 | VGKSTLG |
| Mav-104 DnaB | 118463867 | 1-232 570-795 | 233-569 | S570 | VGKSTLG |
| Mav-PT DnaB | 41394517 | 1-232 650-875 | 233-649 | S650 | VGKSTLG |
| Mbo Pps1 | 31792655 | 1-252 612-846 | 253-611 | C612 | VEGCTAP |
| Mbo RecA | 31619503 | 1-251 692-790 | 252-691 | C692 | KNKCSPP |
| Mbo SufB (Mbo Pps1) | 121637391 | 1-252 612-846 | 253-611 | C612 | VEGCTAP |
| Mbo-1173P DnaB | 121491615 | 1-399 816-874 | 400-815 | S816 | ESGSLEQ |
| Mbo-AF2122 DnaB | 31791235 | 1-399 816-874 | 400-815 | S816 | ESGSLEQ |
| Mca MupF | 53756694 | 1-183 498-765 | 184-497 | C498 | GFNCRCR |
| Mca RIR1 | 53757105 | 1-282 663-1317 | 283-662 | C663 | TNPCAEQ |
| Mch RecA | 10241730 | 1-9 374-423 | 10-373 | S374 | MFGSPET |
| Mex TrbC | 240142173 | 1-144 510-1313 | 145-509 | T510 | AGKTETL |
| Mfa RecA | 10241732 | 1-9 373-422 | 10-372 | S373 | MFGSPET |
| Mfl GyrA | 2501244 | 1-69 491-554 | 70-490 | T491 | MRYTEAR |
| Mfl-ATCC14474 RecA | 10241728 | 1-9 374-423 | 10-373 | S374 | MFGSPET |
| Mfl-PYR-GCK DnaB | 89340273 | 1-236 654-879 | 237-653 | S654 | MGKSTLG |
| Mga GyrA | 11558104 | 1-63 484-550 | 64-483 | T484 | MRYTEAR |
| Mga RecA | 10241734 | 1-9 378-419 | 10-377 | S378 | MFGSPET |
| Mga SufB (Mga Pps1) | 13661022 | 1-15 394-534 | 16-393 | S394 | QYESEVV |
| Mgi-PYR-GCK DnaB | 145221472 | 1-236 654-879 | 237-653 | S654 | MGKSTLG |
| Mgi-PYR-GCK GyrA | 145221415 | 1-130 552-1261 | 131-551 | T552 | MRYTEAR |
| Mgo GyrA | 2501245 | 1-66 487-550 | 67-486 | T487 | MRYTEAR |
| Min DnaB | 11127927 | 1-15 351-576 | 16-350 | S351 | VGKSTLG |
| Mkas GyrA | 2501246 | 1-65 486-549 | 66-485 | T486 | MRYTEAR |
| Mle DnaB | 2959407 | 1-233 379-604 | 234-378 | S379 | VGKSTLG |
| Mle GyrA | 13638291 | 1-130 551-1273 | 131-550 | T551 | MRYTEAR |
| Mle RecA | 480544 | 1-205 571-711 | 206-570 | S571 | MFGSPET |
| Mle SufB (Mle Pps1) | 15827239 | 1-201 588-869 | 202-587 | S588 | SGGSFIY |
| Mma GyrA | 2546990 | 1-49 470-473 | 50-469 | T470 | MRYTEAR |
| MP-Aaphi23 MupF | 31544030 | 1-256 582-800 | 257-581 | C582 | DFNCRCV |
| MP-Be DnaB | 40769425 | 1-140 482-903 | 141-481 | T482 | QDQTKNT |
| MP-Be gp51 | 40769466 | 1-94 412-572 | 95-411 | S412 | NHDSRAR |
| MP-Catera gp206 | 109393388 | 1-149 481-587 | 150-480 | T481 | ELKTQNS |
| MP-Mcjw1 DnaB | 29424612 | 1-92 424-887 | 93-423 | T424 | NGKTELL |
| MP-Omega DnaB | 29425473 | 1-106 447-850 | 107-446 | T447 | EFKTAVN |
| MP-U2 gp50 | 40769364 | 1-94 412-572 | 95-411 | S412 | NHDSRAR |
| Msh RecA | 10241810 | 1-9 374-423 | 10-373 | S374 | MFGSPET |
| Msm DnaB-1 | 118473168 | 1-234 374-1024 | 235-373 | S374 | VGKSTLG |
| Msm DnaB-2 | 118473168 | 1-540 966-1024 | 541-965 | S966 | ESGSLEQ |
| Msp-KMS DnaB | 92913886 | 1-233 556-782 | 234-555 | S556 | MGKSTLG |
| Msp-KMS GyrA | 92913834 | 1-130 552-1257 | 131-551 | T552 | MRYTEAR |
| Msp-MCS DnaB | 91764116 | 1-233 556-782 | 234-555 | S556 | MGKSTLG |
| Msp-MCS GyrA | 108796988 | 1-130 552-1257 | 131-551 | T552 | MRYTEAR |
| Mthe RecA | 10241812 | 1-9 375-424 | 10-374 | S375 | MFGSPET |
| Mtu SufB (Mtu Pps1) | 6686194 | 1-252 612-846 | 253-611 | C612 | VEGCTAP |
| Mtu-CDC1551 DnaB | 13879107 | 1-399 816-874 | 400-815 | S816 | ESGSLEQ |
| Mtu-F11 DnaB | 148719775 | 1-399 816-874 | 400-815 | S816 | ESGSLEQ |
| Mtu-H37Ra DnaB | 148659818 | 1-399 816-874 | 400-815 | S816 | ESGSLEQ |
| Mtu-H37Rv DnaB | 3250719 | 1-399 816-874 | 400-815 | S816 | ESGSLEQ |
| Mtu-H37Rv RecA | 132229 | 1-251 692-790 | 252-691 | C692 | KNKCSPP |
| Mtu-So93 RecA | 2598002 | 1-251 692-790 | 252-691 | C692 | KNKCSPP |
| Mvan DnaB | 90199132 | 1-221 638-863 | 222-637 | S638 | MGKSTLG |
| Mvan GyrA | 119953853 | 1-130 552-1262 | 131-551 | T552 | MRYTEAR |
| Mxa RAD25 | 108762466 | 1-171 608-938 | 172-607 | T608 | GGKTMLA |
| Mxe GyrA | 3023910 | 1-65 264-327 | 66-263 | T264 | MRYTEAP |
| Nfa DnaB | 54027550 | 1-400 826-885 | 401-825 | S826 | ESGSLEQ |
| Nfa Nfa15250 | 54023492 | 1-197 511-643 | 198-510 | C511 | HPNCNCR |
| Nfa RIR1 | 54026278 | 1-156 566-1102 | 157-565 | C566 | PVSCFLL |
| Npu DnaB | 23129113 | 1-388 818-876 | 389-817 | S818 | ESGSIEQ |
| Npu GyrB | 23125273 | 1-77 400-715 | 78-399 | C400 | DIGCGMS |
| Nsp-JS614 DnaB | 71365763 | 1-246 599-824 | 247-598 | S599 | MGKSTLA |
| Nsp-JS614 TOPRIM | 71156494 | 1-61 374-932 | 62-373 | C374 | FFHCFGC |
| Nsp-PCC7120 DnaB | 17228074 | 1-388 818-879 | 389-817 | S818 | ESGSIEQ |
| Nsp-PCC7120 RIR1 | 17231527 | 1-275 683-1172 | 276-682 | S683 | IRRSAGM |
| Plut RIR1 | 78166362 | 1-453 824-1521 | 454-823 | C824 | SNPCSEY |
| Pna RIR1 | 84713443 | 1-297 672-1184 | 298-671 | C672 | TNPCGEQ |
| Pnuc DnaB | 145588611 | 1-238 591-831 | 239-590 | T591 | MGKTAFA |
| Posp-JS666 DnaB | 54029873 | 1-234 586-821 | 235-585 | T586 | MGKTALA |
| Posp-JS666 RIR1 | 54032920 | 1-297 678-1189 | 298-677 | C678 | TNPCGEQ |
| PP-PhiEL Helicase | 82658004 | 1-151 414-940 | 152-413 | T414 | SGKTFTS |
| PP-PhiEL ORF11 | 82657849 | 1-181 495-1088 | 182-494 | T495 | QGKTVSV |
| PP-PhiEL ORF39 | 82657877 | 1-246 613-1036 | 247-612 | S613 | AGKSLTN |
| PP-PhiEL ORF40 | 82657878 | 1-144 393-760 | 145-392 | T393 | FGKTYMA |
| Psy Fha | 28853642 | 1-5986 6135-6274 | 5987-6134 | T6135 | GPCTGPC |
| Rce RIR1 | 209960796 | 1-280 658-1191 | 281-657 | C658 | TNPCGEQ |
| Rma DnaB | 268315593 | 1-425 854-945 | 426-853 | S854 | ESGSIEQ |
| Rsp RIR1 | 85704837 | 1-265 638-1131 | 266-637 | C638 | TNPCGEQ |
| SaP-SETP12 dpol | 126015338 | 1-447 747-1032 | 448-746 | C747 | GKACELG |
| SaP-SETP3 dpol | 125631936 | 1-447 747-1032 | 448-746 | C747 | GKACELG |
| SaP-SETP3 Helicase | 125631932 | 1-33 381-821 | 34-380 | T381 | SGKTSST |
| SaP-SETP5 dpol | 126015336 | 1-447 747-1032 | 448-746 | C747 | GKACELG |
| Sare DnaB | 159040545 | 1-408 785-844 | 409-784 | S785 | ESGSIEQ |
| Sav RecG Helicase | 29606319 | 1-310 649-1075 | 311-648 | T649 | SGKTMVA |
| Sel-PC6301 RIR1 | 56750075 | 1-409 780-1148 | 410-779 | C780 | LNPCGEI |
| Sel-PC7942 RIR1 | 45513096 | 1-409 780-1137 | 410-779 | C780 | LNPCGEI |
| Sep RIR1 | 57867420 | 1-377 762-969 | 378-761 | C762 | SNLCTEI |
| ShP-Sfv-5 Primase | 110616113 | 1-371 714-968 | 372-713 | T714 | HGKTEVV |
| Spl DnaX | 28200485 | 1-129 266-768 | 130-265 | C266 | IDECHML |
| Sru DnaB | 83756970 | 1-230 575-850 | 231-574 | T575 | MGKTAFA |
| Sru PolBc | 83814184 | 1-498 857-1162 | 499-856 | S857 | LINSFYG |
| Sru RIR1 | 83816554 | 1-259 632-932 | 260-631 | C632 | SNLCSEI |
| Ssp DnaB | 2833462 | 1-380 810-872 | 381-809 | S810 | ESGSIEQ |
| Ssp DnaX | 7469304 | 1-129 560-1116 | 130-559 | C560 | IDECHML |
| Ssp GyrB | 2501296 | 1-436 872-1078 | 437-871 | S872 | AGGSAKQ |
| Ssp-JA2 DnaB | 86608559 | 1-388 530-591 | 389-529 | S530 | ESGSIEQ |
| Ssp-JA2 RIR1 | 86608218 | 1-410 780-1137 | 411-779 | C780 | LNPCGEI |
| Ssp-JA3 DnaB | 86606214 | 1-388 530-591 | 389-529 | S530 | ESGSIEQ |
| Ssp-JA3 RIR1 | 86606809 | 1-423 793-1155 | 424-792 | C793 | LNPCGEI |
| StP-Twort ORF6 | 62637191 | 1-147 568-1004 | 148-567 | T568 | GGKTECA |
| Susp-NBC371 DnaB intein | 151425570 | 1-205 550-822 | 206-549 | T550 | MGKTALV |
| Ter DnaB-1 | 110166212 | 1-212 2199-2605 | 213-2198 | T2199 | MGKTSFA |
| Ter DnaB-2 | 113475164 | 1-2366 2544-2605 | 2367-2543 | S2544 | ESGSIEQ |
| Ter DnaE-1 | 110166605 | 1-719 2085-2684 | 720-2084 | C2085 | GVLCYQE |
| Ter DnaE-2 | 110166605 | 1-2110 2539-2684 | 2111-2538 | C2539 | LRRCMGK |
| Ter GyrB | 48893661 | 1-439 684-891 | 440-683 | S684 | ASGSAKQ |
| Ter Ndse-1 | 48891317 | 1-141 478-1080 | 142-477 | C478 | IRSCYDE |
| Ter Ndse-2 | 48891317 | 1-508 939-1080 | 509-938 | T939 | IFNTYGP |
| Ter RIR1-1 | 48890619 | 1-21 416-1985 | 22-415 | S416 | IRRSAGI |
| Ter RIR1-2 | 113474297 | 1-547 921-1985 | 548-920 | C921 | LNPCGEI |
| Ter RIR1-3 | 113474297 | 1-930 1254-1985 | 931-1253 | C1254 | NFHCNLS |
| Ter RIR1-4 | 113474297 | 1-1383 1765-1985 | 1384-1764 | T1765 | PSGTKSL |
| Ter Snf2 | 48894083 | 1-610 1080-1531 | 611-1079 | T1080 | LGKTIQT |
| Ter ThyX | 48891468 | 1-11 310-458 | 12-309 | S310 | IGCSFDV |
| Tfus Hyp-2914 | 72163313 | 1-71 413-659 | 72-412 | S413 | IGYSFGA |
| Tfus RecA-1 | 72161207 | 1-95 518-1177 | 96-517 | T518 | SGKTTVA |
| Tfus RecA-2 | 72161207 | 1-649 1007-1177 | 650-1006 | S1007 | MFGSPET |
| Tth-HB27 DnaE-1 | 46200108 | 1-767 1191-2067 | 768-1190 | S1191 | LRRSMGK |
| Tth-HB27 DnaE-2 | 46200108 | 1-1238 1662-2067 | 1239-1661 | S1662 | FNKSHAA |
| Tth-HB27 RIR1-1 | 46197860 | 1-298 737-1800 | 299-736 | T737 | QGGTRRG |
| Tth-HB27 RIR1-2 | 46197860 | 1-885 1293-1800 | 886-1292 | C1293 | TNPCGEI |
| Tth-HB8 DnaE-1 | 55980149 | 1-767 1191-2067 | 768-1190 | S1191 | LRRSMGK |
| Tth-HB8 DnaE-2 | 55980149 | 1-1238 1662-2067 | 1239-1661 | S1662 | FNKSHAA |
| Tth-HB8 RIR1-1 | 55771457 | 1-300 739-2202 | 301-738 | T739 | QGGTRRG |
| Tth-HB8 RIR1-2 | 55771457 | 1-887 1295-2202 | 888-1294 | C1295 | TNPCGEI |
| Tye RNR-1 | 206742694 | 1-218 596-1385 | 219-595 | S596 | QGESAGA |
| Tye RNR-2 | 206742694 | 1-643 960-1385 | 644-959 | T960 | GFQTPFS |
| Ape APE0745 | 7515990 | 1-175 644-726 | 176-643 | S644 | TTQSAFG |
| Cme-boo Pol-II | 154149675 | 1-886 1052-1307 | 887-1051 | C1052 | RRNCDGD |
| Fac-Fer1 RIR1 | 22405605 | 1-437 804-866 | 438-803 | C804 | TNPCGEQ |
| Fac-Fer1 SufB (Fac Pps1) | 22406063 | 1-245 602-838 | 246-601 | C602 | IEGCTAP |
| Fac-TypeI RIR1 | 257076070 | 1-437 804-1136 | 438-803 | C804 | TNPCGEQ |
| Fac-typeI SufB (Fac Pps1) | 257075583 | 1-242 599-835 | 243-598 | C599 | IEGCTAP |
| Hma CDC21 | 55379381 | 1-331 809-1175 | 332-808 | S809 | TGKSQML |
| Hma Pol-II | 55379319 | 1-965 1146-1395 | 966-1145 | C1146 | RRNCDGD |
| Hma PolB | 55231300 | 1-630 1040-1388 | 631-1039 | S1040 | IMNSLYG |
| Hma TopA | 55231139 | 1-492 1030-1364 | 493-1029 | S1030 | GTKSTRH |
| Hsp-NRC1 CDC21 | 10581599 | 1-282 465-831 | 283-464 | S465 | TGKSQMI |
| Hsp-NRC1 Pol-II | 10581749 | 1-925 1121-1370 | 926-1120 | C1121 | RRNCDGD |
| Hsa-R1 MCM | 169236761 | 1-330 513-879 | 331-512 | S513 | TGKSQMI |
| Hvo PolB | 48927397 | 1-655 1093-1365 | 656-1092 | T1093 | YGDTDSV |
| Hwa GyrB | 110668558 | 1-433 649-852 | 434-648 | S649 | AGGSAKQ |
| Hwa MCM-1 | 109627259 | 1-335 545-2216 | 336-544 | S545 | TGKSAML |
| Hwa MCM-2 | 109627259 | 1-571 1093-2216 | 572-1092 | T1093 | AGLTAAA |
| Hwa MCM-3 | 109627259 | 1-1131 1343-2216 | 1132-1342 | S1343 | KMRSEDR |
| Hwa MCM-4 | 109627259 | 1-1356 1929-2216 | 1357-1928 | S1929 | EQQSISV |
| Hwa Pol-II-1 | 109627078 | 1-966 1496-2289 | 967-1495 | C1496 | RRNCDGD |
| Hwa Pol-II-2 | 109627078 | 1-1524 2069-2289 | 1525-2068 | S2069 | RGGSMDA |
| Hwa PolB-1 | 109625409 | 1-318 753-2142 | 319-752 | C753 | GWNCDDF |
| Hwa PolB-2 | 109625409 | 1-1031 1390-2142 | 1032-1389 | S1390 | IMNSLYG |
| Hwa PolB-3 | 109625409 | 1-1437 1869-2142 | 1438-1868 | T1869 | YGDTDSV |
| Hwa RCF | 110669521 | 1-61 385-649 | 62-384 | T385 | VGKTTAA |
| Hwa RIR1-1 | 109626504 | 1-201 610-1909 | 202-609 | S610 | VFQSGGG |
| Hwa RIR1-2 | 109626504 | 1-1036 1475-1909 | 1037-1474 | T1475 | PTGTTSM |
| Hwa rPol A'' | 109627014 | 1-87 302-637 | 88-301 | T302 | TQMTMNT |
| Hwa Top6B | 110668559 | 1-116 609-1304 | 117-608 | S609 | REQSRGQ |
| Maeo Pol-II | 150014267 | 1-913 1416-1665 | 914-1415 | C1416 | RRNCDGD |
| Maeo RFC | 150014437 | 1-136 754-940 | 137-753 | C754 | ILSCNYP |
| Maeo RNR | 150014168 | 1-338 794-1225 | 339-793 | T794 | GGQTVFS |
| Maeo-N3 Helicase | 150013232 | 1-372 897-1265 | 373-896 | S897 | PTLSAGL |
| Maeo-N3 RtcB | 150400572 | 1-95 581-963 | 96-580 | C581 | DINCGVR |
| Maeo-N3 UDP GP | 145643717 | 1-260 724-906 | 261-723 | C724 | GGSCFPK |
| Memar MCM2 | 125861989 | 1-414 773-1059 | 415-772 | S773 | EQQSISV |
| Memar Pol-II | 125862858 | 1-870 1036-1285 | 871-1035 | C1036 | RRNCDGD |
| Mhu Pol-II | 88189139 | 1-872 1038-1286 | 873-1037 | C1038 | RRNCDGD |
| Mja GF-6P | 2127939 | 1-74 574-1102 | 75-573 | S574 | IGHSRWA |
| Mja Helicase | 15669311 | 1-337 839-1195 | 338-838 | S839 | PTLSAGL |
| Mja Hyp-1 | 1498803 | 1-128 521-785 | 129-520 | C521 | PAHCFTP |
| Mja IF2 | 2495884 | 1-30 577-1155 | 31-576 | T577 | HGKTTLL |
| Mja KlbA | 2833586 | 1-404 573-721 | 405-572 | C573 | HDGCSGT |
| Mja PEP | 2499461 | 1-410 823-1188 | 411-822 | C823 | GLTCHAA |
| Mja Pol-1 | 3915679 | 1-425 795-1634 | 426-794 | S795 | DFRSLYP |
| Mja Pol-2 | 2127863 | 1-882 1359-1634 | 883-1358 | S1359 | LANSVYG |
| Mja r-Gyr | 2129239 | 1-866 1361-1613 | 867-1360 | C1361 | LGLCTYH |
| Mja RFC-1 | 2129234 | 1-53 602-1847 | 54-601 | T602 | VGKTTAA |
| Mja RFC-2 | 2129234 | 1-626 1063-1847 | 627-1062 | S1063 | LNASDER |
| Mja RFC-3 | 2129234 | 1-1124 1668-1847 | 1125-1667 | C1668 | ILSCNYP |
| Mja RNR-1 | 2496131 | 1-337 791-1750 | 338-790 | T791 | GGQTIFS |
| Mja RNR-2 | 2129243 | 1-1058 1592-1750 | 1059-1591 | T1592 | AESTAGR |
| Mja rPol App | 2127865 | 1-75 547-859 | 76-546 | T547 | TQMTMRT |
| Mja rPol A' | 2127864 | 1-463 916-1345 | 464-915 | C916 | LCVCPPY |
| Mja RtcB (Mja Hyp-2) | 2501617 | 1-97 586-968 | 98-585 | C586 | DINCGVR |
| Mja TFIIB | 2129309 | 1-99 435-673 | 100-434 | T435 | MTYTIHD |
| Mja UDP GD | 2129344 | 1-260 715-895 | 261-714 | C715 | GGSCFPK |
| Mka CDC48 | 19886871 | 1-634 1029-1249 | 635-1028 | S1029 | VGESEKK |
| Mka EF2 | 19887115 | 1-34 557-1257 | 35-556 | T557 | HGKTTLS |
| Mka RFC | 19886265 | 1-82 388-635 | 83-387 | S388 | LNASDER |
| Mka RtcB | 19888413 | 1-100 583-988 | 101-582 | C583 | DINCGVR |
| Mka VatB | 19888400 | 1-260 778-990 | 261-777 | C778 | TNYCEAL |
| Mth RIR1 | 7482813 | 1-265 400-703 | 266-399 | C400 | TNPCGEQ |
| Nph CDC21 | 76559046 | 1-331 671-1037 | 332-670 | S671 | TGKSQLL |
| Nph PolB-1 | 76801404 | 1-518 920-1740 | 519-919 | S920 | DLKSLYP |
| Nph PolB-2 | 76801404 | 1-1006 1416-1740 | 1007-1415 | S1416 | IMNSLYG |
| Nph rPol A'' | 76556578 | 1-77 570-894 | 78-569 | T570 | TQMTMNT |
| Pab CDC21-1 | 5458858 | 1-334 499-1112 | 335-498 | S499 | VAKSQLL |
| Pab CDC21-2 | 5458858 | 1-525 794-1112 | 526-793 | T794 | AGLTAAV |
| Pab IF2 | 7521761 | 1-20 415-992 | 21-414 | T415 | HGKTTLL |
| Pab KlbA | 7521679 | 1-453 650-814 | 454-649 | C650 | HDGCMGT |
| Pab Lon | 5459000 | 1-220 554-998 | 221-553 | S554 | PFQSGGL |
| Pab Moaa | 7519988 | 1-155 611-1042 | 156-610 | C611 | CWYCFFY |
| Pab Pol-II | 7514786 | 1-954 1140-1455 | 955-1139 | C1140 | RRNCDGD |
| Pab RFC-1 | 7514225 | 1-61 561-1437 | 62-560 | T561 | VGKTTAA |
| Pab RFC-2 | 7514225 | 1-647 1256-1437 | 648-1255 | C1256 | ILSCNYS |
| Pab RIR1-1 | 7521620 | 1-301 701-2122 | 302-700 | T701 | GGGTGLN |
| Pab RIR1-2 | 7521620 | 1-722 1161-2122 | 723-1160 | S1161 | GAASGPV |
| Pab RIR1-3 | 7521620 | 1-1297 1680-2122 | 1298-1679 | C1680 | TNPCGEE |
| Pab RtcB (Pab Hyp-2) | 7517838 | 1-97 534-916 | 98-533 | C534 | DINCGVR |
| Pab VMA | 7436316 | 1-240 670-1017 | 241-669 | T670 | SGKTVTQ |
| Par RIR1 | 145591871 | 1-439 832-1240 | 440-831 | C832 | VNPCAET |
| Pfu CDC21 | 18892459 | 1-361 729-1049 | 362-728 | T729 | AGLTAAA |
| Pfu IF2 | 18893214 | 1-19 407-984 | 20-406 | T407 | HGKTTLL |
| Pfu KlbA | 18893047 | 1-463 986-1150 | 464-985 | C986 | HDGCMGT |
| Pfu Lon | 18892440 | 1-203 605-1028 | 204-604 | S605 | PFQSGGL |
| Pfu RFC | 18976465 | 1-59 585-852 | 60-584 | T585 | VGKTTAA |
| Pfu RIR1-1 | 1688292 | 1-301 756-1740 | 302-755 | T756 | GGGTGLN |
| Pfu RIR1-2 | 1688292 | 1-914 1297-1740 | 915-1296 | C1297 | TNPCGEE |
| Pfu RtcB (Pfu Hyp-2) | 18893765 | 1-105 587-970 | 106-586 | C587 | DINCGVR |
| Pfu TopA | 18976866 | 1-314 688-1060 | 315-687 | C688 | KGFCSYP |
| Pfu VMA | 18892111 | 1-240 666-1013 | 241-665 | T666 | SGKTVTQ |
| Pho CDC21-1 | 3257012 | 1-334 503-1108 | 335-502 | S503 | VAKSQLL |
| Pho CDC21-2 | 3257012 | 1-529 790-1108 | 530-789 | T790 | AGLTAAV |
| Pho IF2 | 6016319 | 1-22 467-1044 | 23-466 | T467 | HGKTTLL |
| Pho KlbA | 7518763 | 1-451 972-1136 | 452-971 | C972 | HDGCMGT |
| Pho LHR | 3257526 | 1-346 822-1352 | 347-821 | S822 | AVVSSTS |
| Pho Lon | 3256855 | 1-210 685-1127 | 211-684 | S685 | PFQSGGL |
| Pho Pol I | 3913526 | 1-492 953-1235 | 493-952 | S953 | LANSYYG |
| Pho Pol-II | 7518477 | 1-954 1121-1434 | 955-1120 | C1121 | RRNCDGD |
| Pho r-Gyr | 3257210 | 1-953 1364-1624 | 954-1363 | C1364 | AGLCTYH |
| Pho RadA | 7521024 | 1-152 325-529 | 153-324 | T325 | SGKTQLA |
| Pho RFC | 7521348 | 1-61 587-855 | 62-586 | T587 | VGKTTAA |
| Pho RIR1 | 3256754 | 1-467 853-1291 | 468-852 | C853 | TNPCGEE |
| Pho RtcB (Pho Hyp-2) | 14591379 | 1-97 488-871 | 98-487 | C488 | DINCGVR |
| Pho VMA | 3258419 | 1-240 617-964 | 241-616 | T617 | SGKTVTQ |
| Psp-GBD Pol | 2494186 | 1-492 1030-1312 | 493-1029 | S1030 | LANSYYG |
| Pto VMA | 48477562 | 1-236 570-922 | 237-569 | T570 | SGKTVIQ |
| Smar 1471 | 126015182 | 1-377 858-1060 | 378-857 | T858 | AMATGHG |
| Smar MCM2 | 126014943 | 1-429 780-1047 | 430-779 | T780 | EQQTVSI |
| Tac-ATCC25905 VMA | 9502270 | 1-235 409-763 | 236-408 | T409 | SGKTVIQ |
| Tac-DSM1728 VMA | 10639151 | 1-235 410-764 | 236-409 | T410 | SGKTVIQ |
| Tag Pol-1 (Tsp-TY Pol-1) | 2293389 | 1-409 770-1829 | 410-769 | S770 | DFRSLYP |
| Tag Pol-2 (Tsp-TY Pol-2) | 2293389 | 1-854 1393-1829 | 855-1392 | S1393 | LANSYYG |
| Tag Pol-3 (Tsp-TY Pol-3) | 2293389 | 1-1441 1599-1829 | 1442-1598 | T1599 | YADTDGF |
| Tba Pol-II | 197629280 | 1-951 1427-1756 | 952-1426 | C1427 | RRNCDGD |
| Tfu Pol-1 | 3913528 | 1-406 767-1523 | 407-766 | S767 | DFRSLYP |
| Tfu Pol-2 | 3913528 | 1-900 1290-1523 | 901-1289 | T1290 | YADTDGF |
| Thy Pol-1 | 11602745 | 1-458 996-1668 | 459-995 | S996 | LANSYYG |
| Thy Pol-2 | 11602745 | 1-1044 1434-1668 | 1045-1433 | T1434 | YADTDGF |
| Tko CDC21-1 | 57641555 | 1-362 503-1157 | 363-502 | T503 | AGLTAAA |
| Tko CDC21-2 | 57641555 | 1-553 889-1157 | 554-888 | T889 | EQQTISI |
| Tko Helicase | 57641267 | 1-331 735-1125 | 332-734 | S735 | PTLSAGI |
| Tko IF2 | 57641240 | 1-20 567-1144 | 21-566 | T567 | HGKTTLL |
| Tko KlbA | 57641788 | 1-511 1035-1198 | 512-1034 | C1035 | HNGCMGT |
| Tko LHR | 57640699 | 1-345 871-1390 | 346-870 | S871 | AVVSSTS |
| Tko Pol-1 (Pko Pol-1) | 2129415 | 1-406 767-1670 | 407-766 | S767 | DFRSLYP |
| Tko Pol-2 (Pko Pol-2) | 2129415 | 1-851 1388-1670 | 852-1387 | S1388 | LANSYYG |
| Tko Pol-II | 57641838 | 1-963 1438-1798 | 964-1437 | C1438 | RRNCDGD |
| Tko r-Gyr | 57640405 | 1-960 1450-1711 | 961-1449 | C1450 | AGLCTYH |
| Tko RadA | 57641834 | 1-149 632-836 | 150-631 | T632 | SGKTQLA |
| Tko RFC | 57642153 | 1-59 600-866 | 60-599 | T600 | VGKTTAA |
| Tko RIR1-1 | 57641671 | 1-301 756-1746 | 302-755 | T756 | GGGTGLN |
| Tko RIR1-2 | 57641671 | 1-914 1297-1746 | 915-1296 | C1297 | TNPCGEE |
| Tko TopA | 57641026 | 1-315 827-1229 | 316-826 | C827 | KGYCSYP |
| Tli Pol-1 | 543522 | 1-494 1033-1702 | 495-1032 | S1033 | LANSYYG |
| Tli Pol-2 | 543522 | 1-1081 1472-1702 | 1082-1471 | T1472 | YADTDGF |
| Ton-NA1 Pol | 83338486 | 1-491 1027-1308 | 492-1026 | S1027 | LANSYYG |
| Tsp-GE8 Pol-1 | 10799895 | 1-491 1027-1699 | 492-1026 | S1027 | LANSYYG |
| Tsp-GE8 Pol-2 | 10799895 | 1-1075 1465-1699 | 1076-1464 | T1465 | YADTDGF |
| Tsp-GT Pol-1 | 86753391 | 1-491 1029-1701 | 492-1028 | S1029 | LANSYYG |
| Tsp-GT Pol-2 | 86753391 | 1-1077 1467-1701 | 1078-1466 | T1467 | YADTDGF |
| Tsp-OGL-20P Pol | 117958105 | 1-491 1029-1311 | 492-1028 | S1029 | LANSYYG |
| Tthi Pol | 117958105 | 1-491 1029-1311 | 492-1028 | S1029 | LANSYYG |
| Tvo VMA | 13540883 | 1-235 422-776 | 236-421 | T422 | SGKTVIQ |
| Tzi Pol | 86753389 | 1-491 1024-1467 | 492-1023 | S1024 | LANSYYG |
| Unc-ERS PFL | 52548443 | 1-33 468-666 | 34-467 | C468 | CPYCHNP |
| Unc-ERS RIR1 | 52550429 | 1-289 597-914 | 290-596 | C597 | TNPCGEQ |
| Unc-ERS RNR | 52548271 | 1-152 743-981 | 153-742 | T743 | NAETSHK |
| Unc-MetRFS MCM2 | 110622082 | 1-408 576-862 | 409-575 | S576 | EQQSISI |
